# Machine Learning‐Enhanced Ultrasensitive Immuno‐CRISPR Array Facilitates Early Diagnosis of Alzheimer's Disease by Detecting Multiple Plasma Biomarkers

**DOI:** 10.1002/advs.75983

**Published:** 2026-06-09

**Authors:** Liding Zhang, Changwen Yang, Qian Yao, Xuewei Du, Shuai Ding, Yaoqiang Shi, Can Sheng, Ming Wang, Ying Han, Haiming Luo

**Affiliations:** ^1^ Key Laboratory of Biomedical Engineering of Hainan Province School of Biomedical Engineering Hainan University Haikou China; ^2^ MOE Key Laboratory for Biomedical Photonics Wuhan National Laboratory for Optoelectronics Huazhong University of Science and Technology Wuhan China; ^3^ Department of Clinical Laboratory Renmin Hospital of Wuhan University Wuhan China; ^4^ Institute of Basic and Clinical Medicine The First People's Hospital of Yunnan Kunming China; ^5^ Department of Neurology the Affiliated Hospital of Jining Medical University Jining China; ^6^ Department of Neurology Xuanwu Hospital of Capital Medical University Beijing China

**Keywords:** Alzheimer's disease, early diagnosis, immuno‐CRISPR, multiple RPA, multi‐target detection, plasma biomarkers

## Abstract

Early and accurate diagnosis of Alzheimer's disease (AD) remains a significant challenge due to the multifactorial and dynamic nature of its pathology. Although plasma‐based biomarkers such as amyloid‐β (Aβ) and phosphorylated tau (p‐tau) have shown promise as diagnostic indicators, current single‐biomarker detection techniques lack the requisite sensitivity and specificity for early‐stage diagnosis. Here, we present the development of an ultrasensitive CRISPR‐based multi‐protein detection array (UCMDA) capable of concurrently detecting six core AD biomarkers, including Aβ_40_, Aβ_42_, p‐tau^181^, p‐tau^217^, p‐tau^231^, and p‐tau^396,404^. By integrating antibody pair‐based multiplex recombinase polymerase amplification (RPA) with spatially encoded CRISPR‐Cas12a detection, the UCMDA achieves a detection limit of 1 fg/mL, which is 10 000‐fold more sensitive than conventional ELISA. Clinical validation in a cohort of 155 plasma samples demonstrated that logistic regression (LR)‐based integration of the six biomarkers significantly enhanced diagnostic performance, with the multi‐biomarker model substantially outperforming single‐biomarker approaches in diagnosing AD‐MCI and AD. This platform offers a scalable, cost‐effective, and minimally invasive strategy for early detection and disease monitoring. This work highlights the potential of CRISPR‐based multiplex protein detection technologies combined with machine learning‐assisted analysis to enhance the precision of diagnosing neurodegenerative disorders.

## Graphical abstract

This work presents a CRISPR‐based, ultrasensitive multiplex protein detection array capable of simultaneously analyzing six plasma biomarkers associated with Alzheimer's disease (AD). By integrating antibody‐mediated signal transduction, recombinase polymerase amplification, and spatially encoded CRISPR‐Cas12a, the system achieves detection sensitivity at the femtogram level detection. Furthermore, the incorporation of machine learning techniques to analyze multi‐biomarker signatures further enhances the accuracy of early AD diagnosis, offering a scalable strategy for achieving precise blood‐based diagnostics.

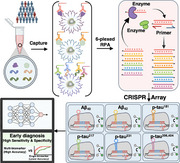

## 1 Introduction

Alzheimer's disease (AD) is the predominant cause of dementia, affecting over 55 million individuals globally and posing a profound challenge to public health systems [1, 2]. The emergence of multiple disease‐modifying therapies has further highlighted the importance of early and accurate diagnosis, as interventions initiated during the prodromal and mild cognitive impairment (MCI) stages have the potential to slow cognitive deterioration and maintain functional autonomy [3, 4]. However, the insidious onset and protracted preclinical phase of AD (decades prior to symptom manifestation) pose significant diagnostic challenges. Currently, the diagnostic framework for AD is anchored in the detection of extracellular amyloid‐β (Aβ) plaques and intracellular neurofibrillary tangles composed of hyperphosphorylated tau (p‐tau) protein [5, 6]. These neuropathological changes occur several years prior to the onset of clinical symptoms, offering a critical window for early intervention. Cerebrospinal fluid (CSF) biomarkers reflecting Aβ and tau pathologies provide a direct biochemical assessment of central nervous system pathology and are considered the gold standard for AD diagnosis [6, 7]. Nonetheless, the invasiveness of lumbar puncture procedures, coupled with the necessity for specialized personnel, limits their feasibility in large‐scale screening and routine clinical application [8]. In contrast, plasma‐based biomarkers have emerged as a transformative approach, providing a minimally invasive, cost‐efficient, and scalable method for detecting AD pathology [9, 10].

Recent advances in ultrasensitive immunoassay technologies, such as single‐molecule arrays (Simoa) and meso‐scale discovery (MSD), have enabled the precise quantification of plasma Aβ_42_ and phosphorylated tau proteins at threonine 181 (p‐tau^181^), threonine 217 (p‐tau^217^), and threonine 231 (p‐tau^231^) [9, 10, 11, 12, 13]. The multifactorial and dynamic nature of AD across different disease stages reduces the diagnostic reliability of single biomarker strategies, especially in the early stages of disease progression, such as AD‐MCI and subjective cognitive decline (SCD) [14, 15]. Tau proteins undergo extensive post‐translational modifications, encompassing more than 50 phosphorylation sites, the temporal patterns, and pathological implications of which have not yet been fully elucidated [16]. In addition, individual isoforms of Aβ and p‐tau exhibit limited disease specificity, as they are also found in various other neurodegenerative disorders, such as Lewy bodies, vascular dementia, Down syndrome, and delirium [17, 18, 19, 20, 21, 22].

These findings highlight the need for multiplexed detection platforms capable of simultaneously measuring a range of AD‐related biomarkers with high sensitivity and specificity. However, existing multi‐biomarker detection methods, such as Simoa, MSD, immunoprecipitation‐mass spectrometry, nucleic acid‐linked immuno‐sandwich assay (NULISA), and Olink, face significant technical and logistical challenges, including high equipment costs, complex operational procedures, and limited scalability for community settings or primary care applications [23, 24, 25]. CRISPR‐based molecular diagnostics have revolutionized nucleic acid detection by offering exceptional sensitivity, specificity, and programmability under isothermal conditions [26, 27, 28, 29, 30]. Recombinase polymerase amplification (RPA) coupled with the CRISPR‐Cas system has demonstrated robust performance in pathogen detection, eliminating the requirement for complex instrumentation and facilitating rapid, on‐site testing [28, 31]. However, the expansion of CRISPR diagnostics to multiplexed protein biomarker assays remains largely unexplored, hindered by difficulties in target recognition, signal transduction, and discrimination, as well as challenges associated with assay scalability.

To explore the potential of the diagnostic and prognostic value of simultaneously measuring different forms of Aβ and p‐tau, we proposed an ultrasensitive CRISPR‐based multi‐protein detection array (UCMDA) capable of simultaneously detecting six plasma biomarkers closely related to AD pathophysiology. By integrating antibody pair‐based multiplex RPA with spatially encoded CRISPR‐Cas12a arrays, our platform achieves femtogram‐level sensitivity and can accurately identify Aβ_42_, Aβ_40_, p‐tau^181^, p‐tau^217^, p‐tau^231^, and p‐tau^396,404^ isoforms in clinical plasma samples. UCMDA addresses critical limitations of current methodologies by providing a scalable, cost‐efficient, and user‐friendly approach, enabling early and accurate AD diagnosis. Furthermore, this study employed logistic regression (LR) to integrate multi‐biomarker data, thereby achieving a robust combination of features and enhancing classification performance. Clinical validation results based on 155 plasma samples demonstrated that this LR‐based multi‐biomarker model significantly outperformed conventional single‐biomarker strategies, highlighting its potential for broad clinical application.

## 2 Results

### 2.1 Design and Operation of UCMDA

**FIGURE 1:**
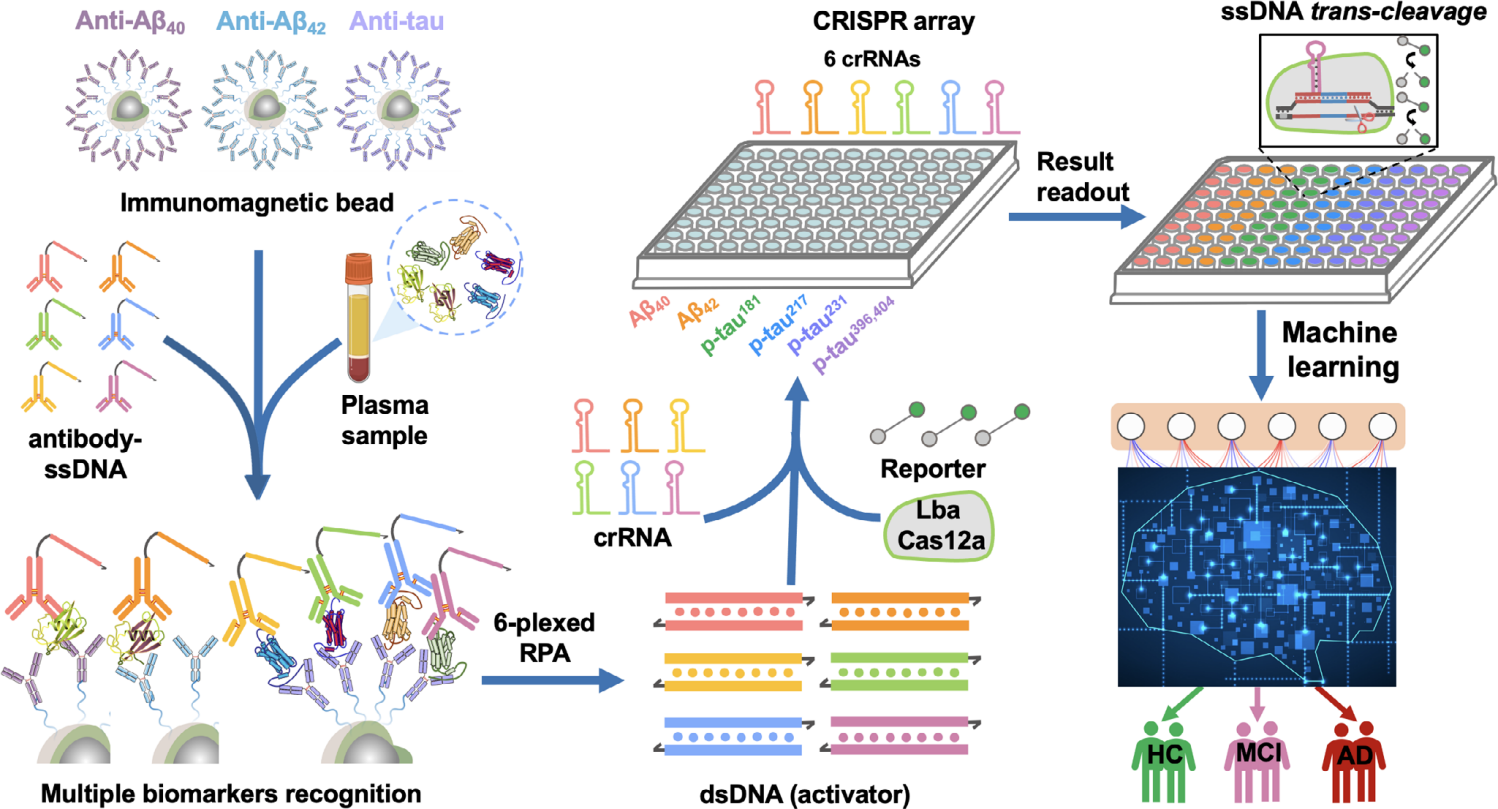
Schematic illustration of UCMDA for the simultaneous detection of multiple AD core biomarkers. Sequence‐specific ssDNA was modified on the detection antibody as the output of the detection signal. UCMDA uses an “amplify together” strategy to simultaneously amplify biomarker signals using antibody pair‐based multiple RPA assay, followed by an “individual detection” principle based on a CRISPR array. The wells in the array were preloaded with different crRNAs that specifically recognize the corresponding dsDNA produced by multiple RPA assay. After CRISPR cleavage, the fluorescent readout at a specific well indicated the level of the relevant biomarker in the sample.

The detection concept of UCMDA is to convert the plasma biomarker detection into several CRISPR‐Cas12a‐targeted double‐stranded DNA (dsDNA) testing so that the indiscriminate single‐stranded DNase activity of Cas12a can be released in the presence of a matching crRNA. As shown in Figure 1, in the presence of antibody‐ssDNA, plasma biomarkers were enriched and formed a sandwich structure of magnetic beads@biomarkers@antibody‐ssDNA. Subsequently, the conjugated antibody‐ssDNA was applied to a multiplex RPA assay for amplifying the biomarker signal. The amplified products were then loaded onto the microarray and analyzed via a CRISPR‐based cleavage assay. Detection of multiple biomarkers relies on a microarray preloaded with a specific CRISPR detection mixture containing Cas12a, crRNA, and a fluorescence reporter. If the sample contains the target biomarker, the amplified dsDNA matches the crRNA, and the corresponding well shows a bright fluorescent signal. Conversely, in the absence of the target biomarker or other non‐target biomarkers, a lower background signal is observed owing to a mismatch between dsDNA and crRNA. Different biomarkers are identified by the corresponding designed crRNA pre‐arranged on the array. Therefore, UCMDA combines antibody‐based multi‐RPA with a CRISPR‐based spatial encoding strategy, following the principle of “amplify together and detect individually” to detect multiple biomarkers accurately and simultaneously.

### 2.2 Characterization of Antibody Pairs for Detecting Different Aβ and p‐tau Biomarkers

**FIGURE 2:**
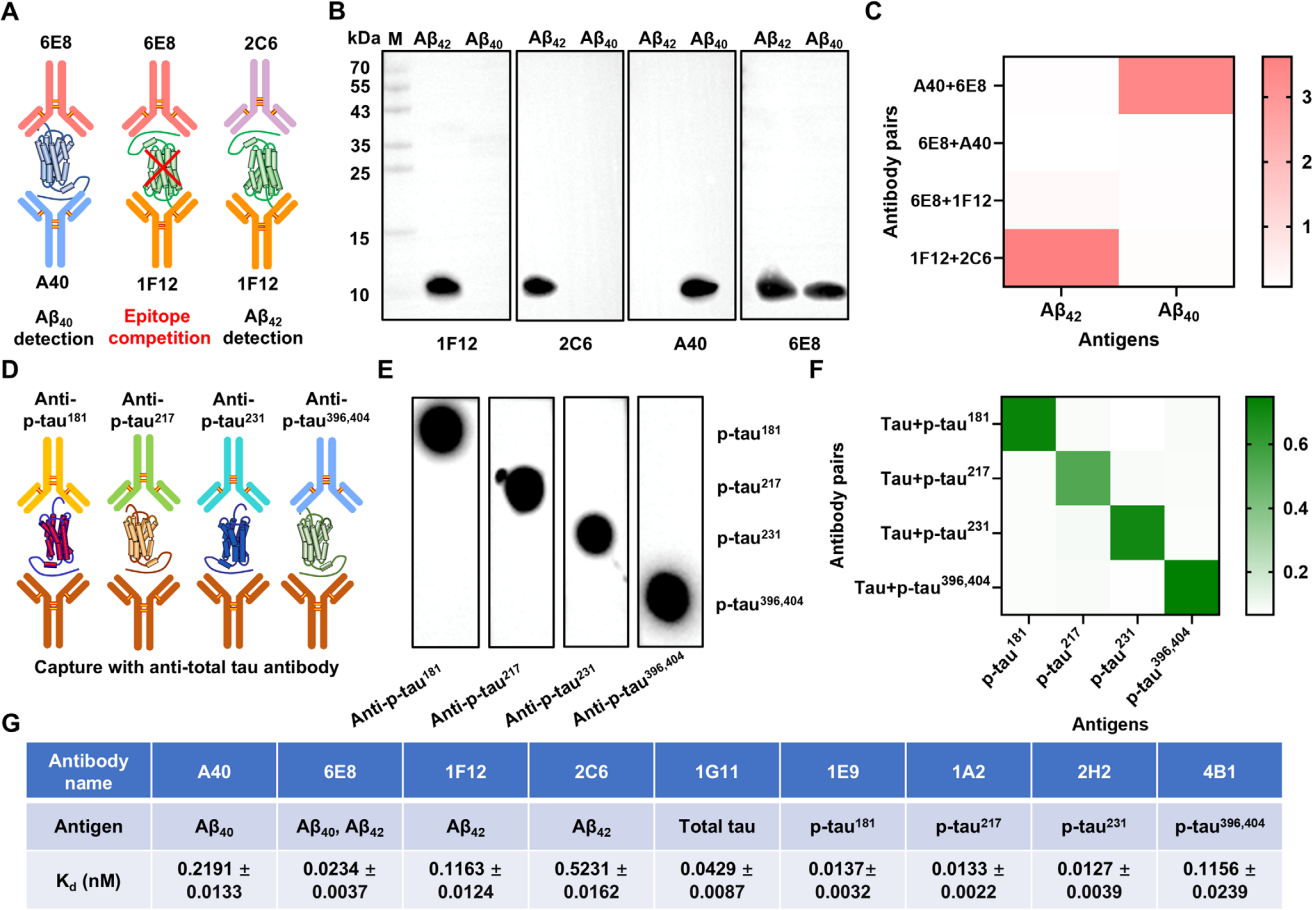
Detection strategies for different species of Aβ and p‐tau biomarkers. (A) Schematic diagram of the detection strategy for Aβ_40_ and Aβ_42_. (B) Western blot analysis of the selectivity of antibodies for Aβ_42_ and Aβ_40_. (C) Heat map analysis of antibody pairs for the ELISA‐based detection of Aβ_42_ and Aβ_40_. (D) Schematic representation of different p‐tau detection strategies. (E) Dot blot analysis of antibody selectivity for different p‐tau proteins. (F) Heat map analysis of antibody pairs for the ELISA‐based detection of different p‐tau proteins. (G) K_d_ values of antibodies against the corresponding antigens.

Sensitive and specific detection of multiple biomarkers in the plasma directly depends on their selective antibody pairs. Thus, to selectively detect Aβ_40_ and Aβ_42_, we selected four Aβ conformation‐dependent antibodies with different epitopes, of which A40/6E8 was used for Aβ_40_ detection and 1F12/2C6 was used for Aβ_42_ detection (Figure 2A, B and Figure S1A,B). Of note, there was an epitope competition between 1F12 (3–9 of Aβ) and 6E8 (1–7 of Aβ), preventing the false detection of Aβ_42_ by the 1F12 and 6E8 combination. The results showed that these two antibody pairs could accurately detect Aβ_40_ and Aβ_42_ without cross‐reaction (Figure 2C and Figure S1C). In the detection of different p‐tau proteins, an anti‐total tau antibody was used as the capture antibody to enrich all forms of p‐tau, and specific detection was performed using antibodies against different p‐tau proteins (Figure 2D). Dot blot and ELISA results showed that the antibodies had a specific phosphorylation site selectivity and did not cross‐react with other p‐tau proteins (Figure 2E and Figure S2A,B). ELISA results and heat map analysis confirmed that this detection strategy could specifically detect multiple p‐tau proteins (Figure 2F and Figure S2C). The results of the binding affinity test showed that all antibodies exhibited a strong binding affinity for their corresponding antigens, with K_d_ values as low as picomolar, ensuring rapid recognition in the UCMDA system (Figure 2G).

### 2.3 Specificity of ssDNA, RPA Primers, and crRNA and Their Matching Evaluation

In the UCMDA system, the detection of biomarkers in the plasma is achieved by the detection of ssDNA based on RPA‐CRISPR technology, in which the ssDNA is modified on the detection antibody. Therefore, in addition to the selection of specific antibodies, it is crucial to ensure that the designed ssDNA, RPA primers, and corresponding crRNA are highly selective. We first designed six sequence‐specific ssDNA (Figure S3 and Table S1) and their corresponding RPA primers (Table S2). Subsequently, we evaluated the specificity and efficiency of the designed RPA primers in amplifying the corresponding ssDNA. Agarose gel images after RPA assay showed that the primers were highly specific to the corresponding ssDNA (Figure S4), which ensured the high specificity of RPA.

**FIGURE 3:**
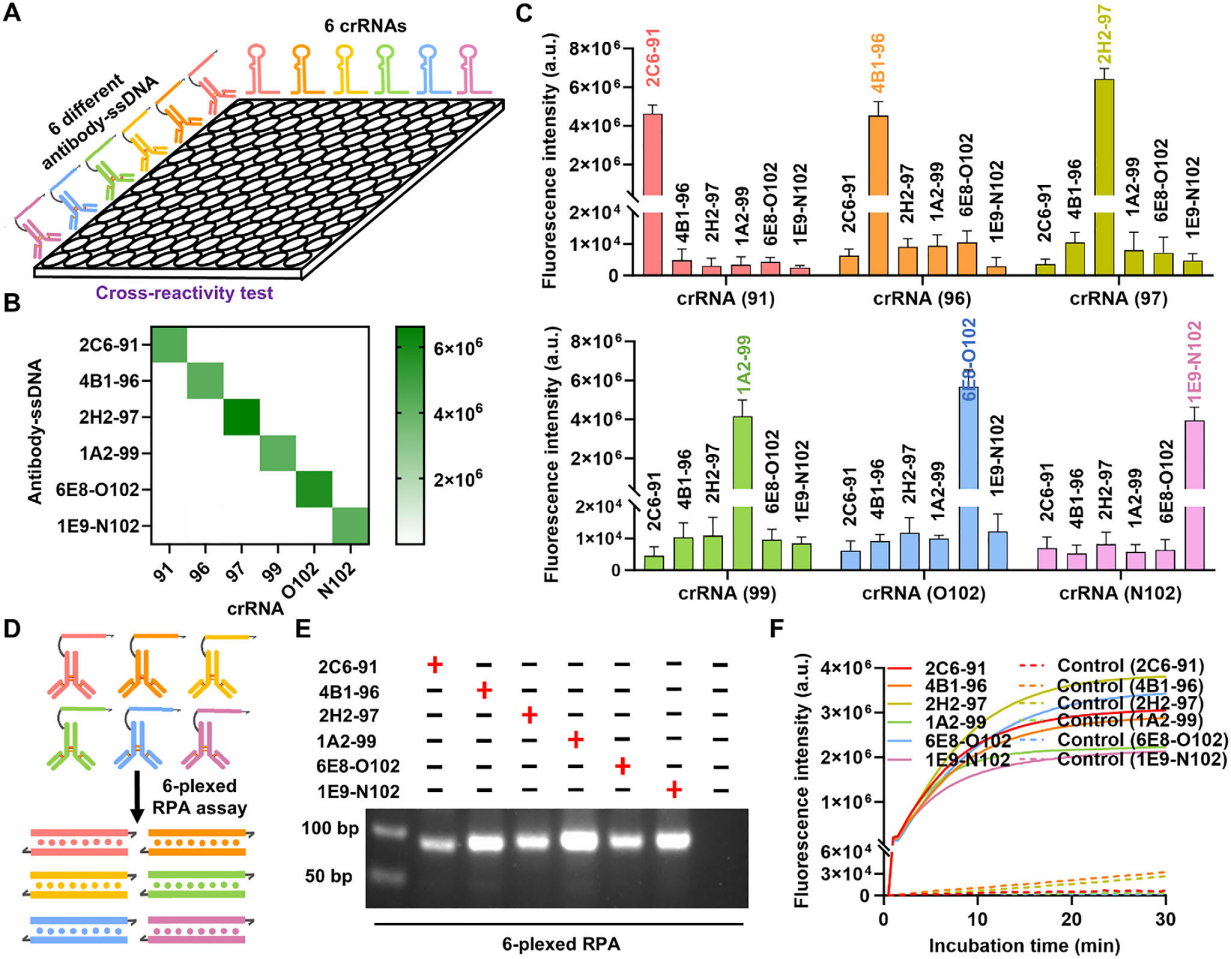
Comprehensive characterization of crRNA pools and 6‐plexed RPA performance. (A) Matrix‐based reactivity test of six crRNAs against six designed ssDNA after RPA assay. (B) Fluorescence‐based test results are shown as a heat map. (C) Quantitative analysis of the reaction of a single crRNA with six different ssDNA. (D) Schematic illustration of 6‐plexed RPA assay. (E) Agarose gel image of the product after 6‐plexed RPA using different antibody‐ssDNA conjugates. (F) Kinetic curves of CRISPR‐Cas12a detection of different antibody‐ssDNA. Data are presented as mean or mean ± s.d. (n = 3).

The crRNA matching each ssDNA was then designed according to the common principles of LbaCas12a design (Table S3). The AAUUUCUACUAAGUGUAGAU sequence was used as a common scaffold sequence for LbaCas12a crRNA (Figure S5A) [32, 33, 34], but the fluorescence intensity was significantly lower than that of the GAAUUUCUACUGUUGUAGAU scaffold sequence under the same reaction conditions (Figure S5B–E). This observation may be attributed to the fact that the free energy of the sequence GAAUUUCUACUGUUGUAGAU was calculated as ‐4.83 kcal/mol, which is higher than that of the common scaffold sequence of AAUUUCUACUAAGUGUAGAU (−5.28 kcal/mol). The relatively low free energy of AAUUUCUACUAAGUGUAGAU requires more energy to change the conformation of the crRNA spacer region from the original state to the “linear” state. Only in this state, the secondary structure of the spacer region opens and prepares to hybridize with the DNA target. Remarkably, this hypothesis was confirmed by Zhang et al. that the secondary structure of crRNA severely impedes hybridization of the two chains, potentially reducing the trans‐ cleavage activity of Cas12a [35]. Following this, a 6 × 6 matrix test (6 crRNA × 6 ssDNA) was designed to verify the recognition specificity of crRNA (Figure 3A). No extensive cross‐activity was observed in this test (Figure 3B). A similar trend was observed when crRNA was analyzed alone (Figure 3C). The fluorescence kinetics of CRISPR‐Cas12a showed that the collateral cleavage activity of CRISPR‐Cas12a was triggered by the targeted dsDNA and rapidly reached a peak within 30 min in the presence of the matched crRNA (Figure S6). These results indicated that the designed highly specific crRNA can be used for a subsequent multiplex CRISPR array.

### 2.4 Validation of 6‐Plexed RPA for the Amplification and Conversion of Biomarker Signals

To convert the detection of plasma biomarkers into the detection of several pre‐designed CRISPR‐Cas12a‐targetable dsDNA, antibody‐ssDNA conjugates were initially synthesized by linking sulfhydryl‐modified ssDNA (ssDNA‐SH) and antibodies with NHS‐dPEG‐Mal, wherein the NHS group of NHS‐dPEG‐Mal reacts with lysine residues of antibodies to form a stable amide bond and the Mal group reacts with ssDNA‐SH to form a stable covalent bond (Figure S7A). The image of polyacrylamide gel showed that the molecular weight of the antibody‐ssDNA conjugate was larger than that of the unmodified antibody (Figure S7B). In addition, the purified antibody‐ssDNA conjugates were identified by PCR, and the agarose gel image showed that six different ssDNAs were successfully introduced to their corresponding antibodies (Figure S7C). ELISA results showed that the biological activity of antibody‐ssDNA was comparable to that of the original antibody, because dPEG provided a flexible linker arm to ensure the biological activity of the antibody and ssDNA (Figure S8).

After synthesizing different antibody‐ssDNA conjugates, we established a 6‐plexed RPA to amplify six different biomarker signals (Figure 3D). As shown in Figure 3E, the corresponding target ssDNA can be accurately amplified in the 6‐plexed RPA system. We next applied the CRISPR‐Cas12a system to detect the products of the 6‐plexed RPA. When crRNA recognizes its target dsDNA, the trans‐cleavage ability of Cas12a is activated (Figure 3F).

### 2.5 Specificity and Sensitivity of UCMDA

**FIGURE 4:**
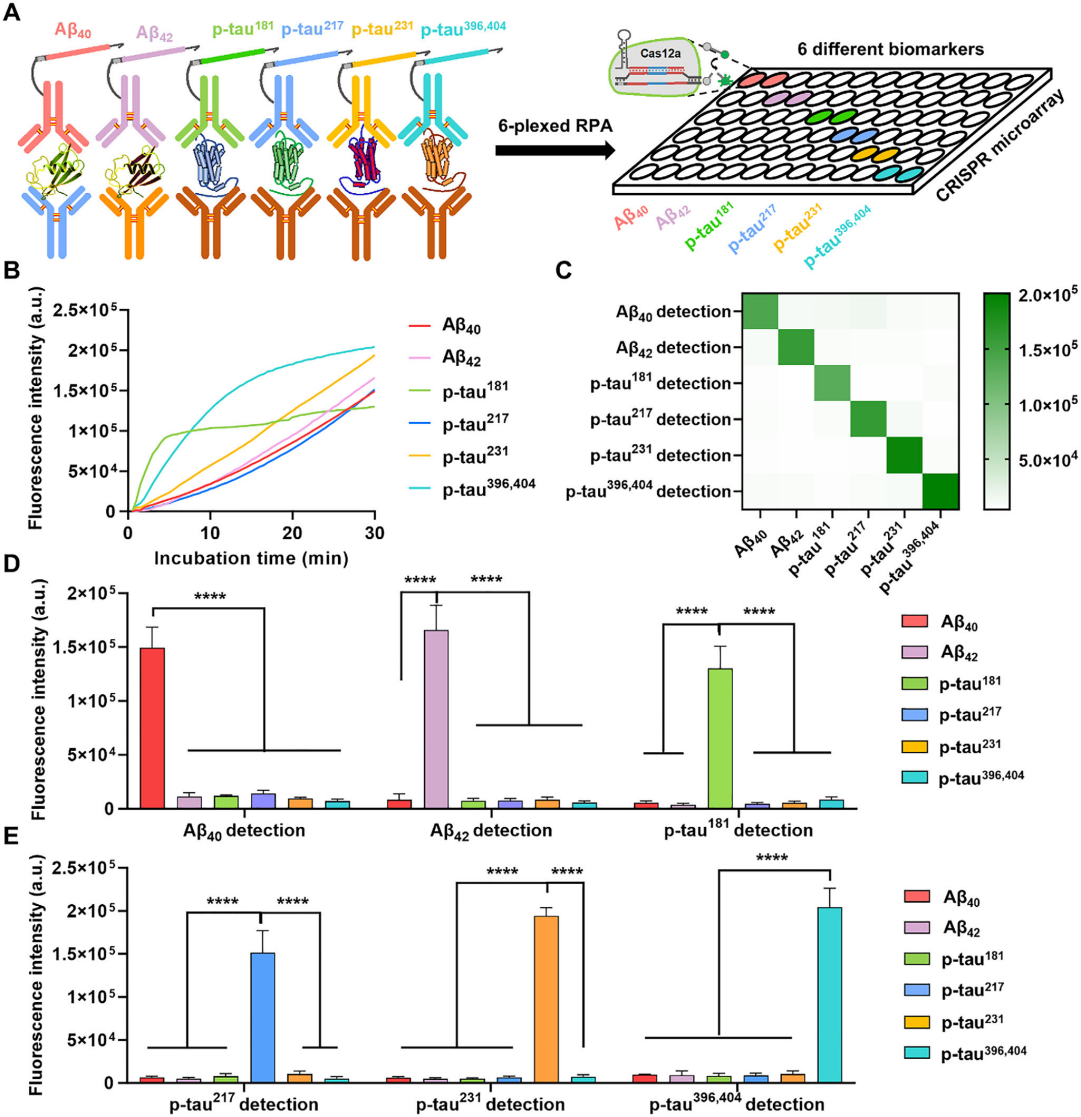
Selectivity of UCMDA in detecting six AD plasma biomarkers. (A) Schematic diagram of the conversion of the plasma biomarker detection to RPA‐CRISPR‐Cas12a‐based nucleic acid testing. (B) Kinetic curves of UCMDA for detecting different Aβ and p‐tau biomarkers. (C‐E) Heat map (C) and quantitative analysis (D, E) of the selectivity of UCMDA for detecting Aβ_40_, Aβ_42_, p‐tau^181^, p‐tau^217^, p‐tau^231^, and p‐tau^396,404^. Data are presented as mean or mean ± s.d. (n = 3, ^****^ *p* ≤ 0.0001). One‐way ANOVA was used for statistical analysis.

UCMDA mainly comprises two parts: 6‐plexed RPA based on antibody pairs and a CRISPR array, as shown in Figure 4A. UCMDA was also determined to be highly specific, as demonstrated by the selective antibody pairs (Figure 2 and Figures S1 and S2), sequence‐specific ssDNA (Figure S3), and matched primers and crRNA (Figure 3 and Figures S4–S6). The specificity of UCMDA for detecting multiple AD biomarkers was further investigated. It performed well in the detection of multiple AD plasma biomarkers, including Aβ_40_, Aβ_42_, p‐tau^181^, p‐tau^217^, p‐tau^231^, and p‐tau^396,404^ (Figure 4B), and showed low fluorescence in non‐targeted biomarker detection. This confirmed the low cross‐reactivity between different Aβ and p‐tau isoforms (Figure 4C). Notably, similar results were observed when the biomarkers were individually analyzed (Figure 4D,E and Figure S9). These results strongly suggest that the established UCMDA can achieve simultaneous and accurate detection of multiple AD plasma biomarkers.

**FIGURE 5:**
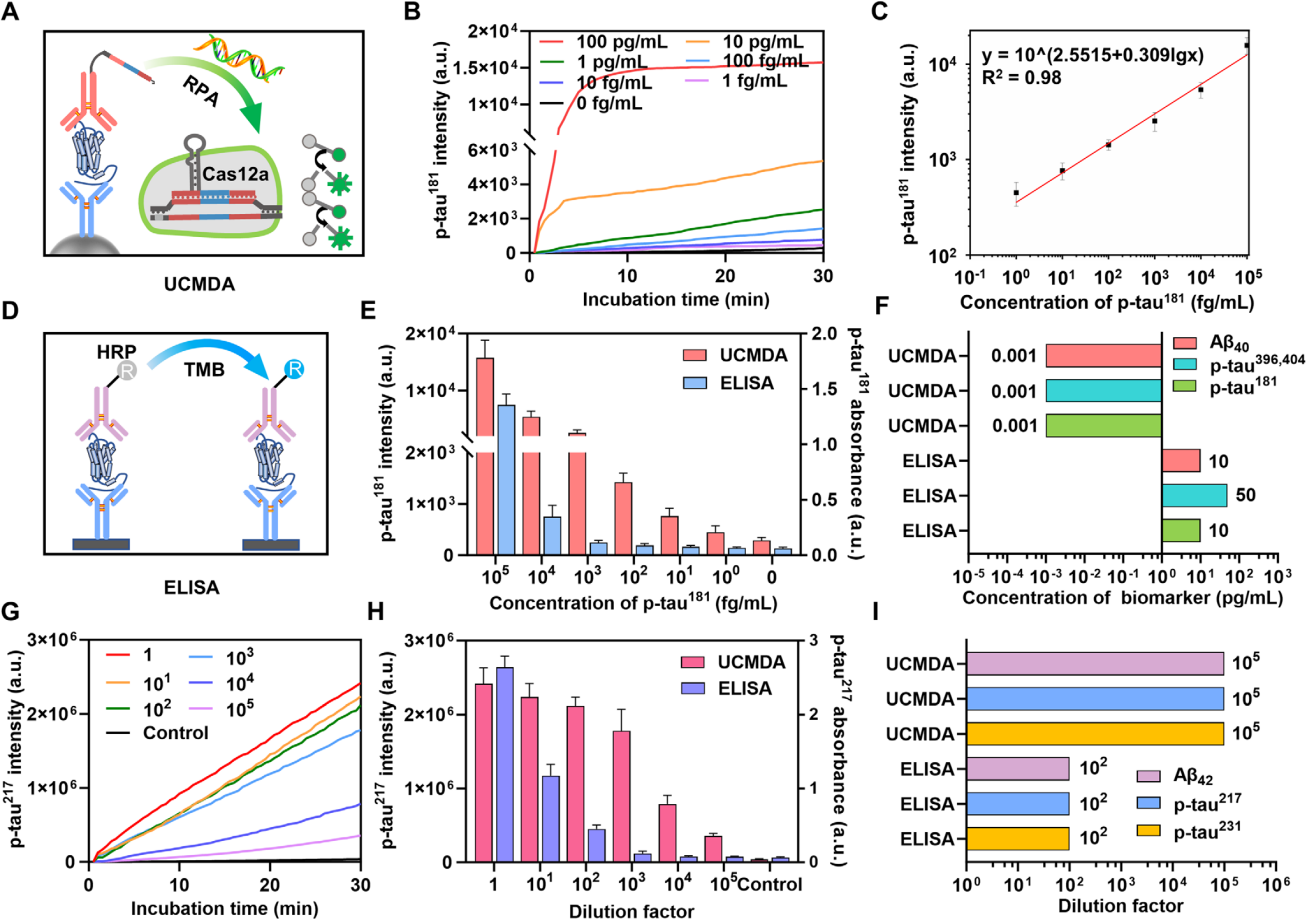
Sensitivity test of UCMDA for six AD plasma biomarkers. (A, D) Schematic diagram of UCMDA (A) and ELISA (D) for biomarker detection. (B, G) Kinetic curves of UCMDA for detecting different concentrations of p‐tau^181^ in TBS buffer (B) and p‐tau^217^ (G) in the plasma of htau mice. (C) Calibration curve of UCMDA for the detection of p‐tau^181^. (E, H) Detection of different concentrations of p‐tau^181^ in TBS buffer (E) and p‐tau^217^ in the plasma of htau mice (H) using UCMDA and standard ELISA. (F) Sensitivity comparison of UCMDA and ELISA for the detection of Aβ_40_, p‐tau^181^, and p‐tau^396,404^ in TBS buffer. (I) Sensitivity comparison of UCMDA and ELISA for the detection of Aβ_42_, p‐tau^217^, and p‐tau^231^ in the plasma of transgenic mice. Data are presented as mean or mean ± s.d. (n = 3).

Subsequently, we evaluated the detection performance of UCMDA for low‐abundance biomarkers. The detection efficacy of UCMDA was assessed using Aβ_40_, p‐tau^181^, and p‐tau^396,404^ as representative examples with varying concentrations of standard protein solutions ranging from 1 fg/mL to 100 pg/mL (Figure 5A). The fluorescence signal of UCMDA responded well to the concentration changes in Aβ_40_, p‐tau^181^, or p‐tau^396,404^ (Figure 5B and Figure S10A, B). The limit of quantitation was 1 fg/mL with a dynamic range from 1 fg/mL to 100 pg/mL (Figure 5C and Figure S10C, D). The sensitivity of UCMDA was further compared with that of standard ELISA at the same biomarker concentrations (Figure 5D). The results of ELISA were only valid for 10 pg/mL Aβ_40_ (Figure S10E), 10 pg/mL p‐tau^181^ (Figure 5E), or 50 pg/mL p‐tau^396,404^ (Figure S10F). This suggested that the detection sensitivity of UCMDA in a buffer environment is approximately 10 000‐fold higher than that of ELISA (Figure 5F). Meanwhile, the sensitivity of UCMDA was further validated using a series of 5×FAD and htau mouse plasma samples with dilution factors ranging from 1 to 100 000. UCMDA could detect Aβ_42_ (Figure S11A), p‐tau^217^ (Figure 5G), and p‐tau^231^ (Figure S11C) across the entire dilution series, but ELISA was only effective at a 100‐fold dilution (Figure 5H and Figure S11B, D), indicating that the UCMDA is at least 1000‐fold more sensitive than ELISA and similar to previously developed assays for plasma sample testing (Figure 5I and Table S4).

### 2.6 Performance Evaluation of UCMDA in Detecting Multiple Biomarkers

The performance of UCMDA in detecting Aβ_40_ and Aβ_42_ in 5×FAD mouse plasma and multiple p‐tau proteins (including p‐tau^181^, p‐tau^217^, p‐tau^231^, and p‐tau^396,404^) in htau mouse plasma was evaluated. Figure S12A shows a schematic diagram of UCMDA for plasma testing. The kinetic curves showed that UCMDA could accurately detect Aβ_40_, Aβ_42_, p‐tau^181^, p‐tau^217^, p‐tau^231^, and p‐tau^396,404^ (Figure S12B–G) in the certified plasma samples of Aβ/p‐tau‐positive mice as per standard ELISA (Figure S13), whereas the fluorescence detected for these biomarkers in the plasma of C57BL/6J mice was very weak. The significant differences in the fluorescence intensity of plasma biomarkers between AD transgenic mice and normal C57BL/6 mice indicated that the UCMDA platform has great potential for early and accurate diagnosis of AD.

**FIGURE 6:**
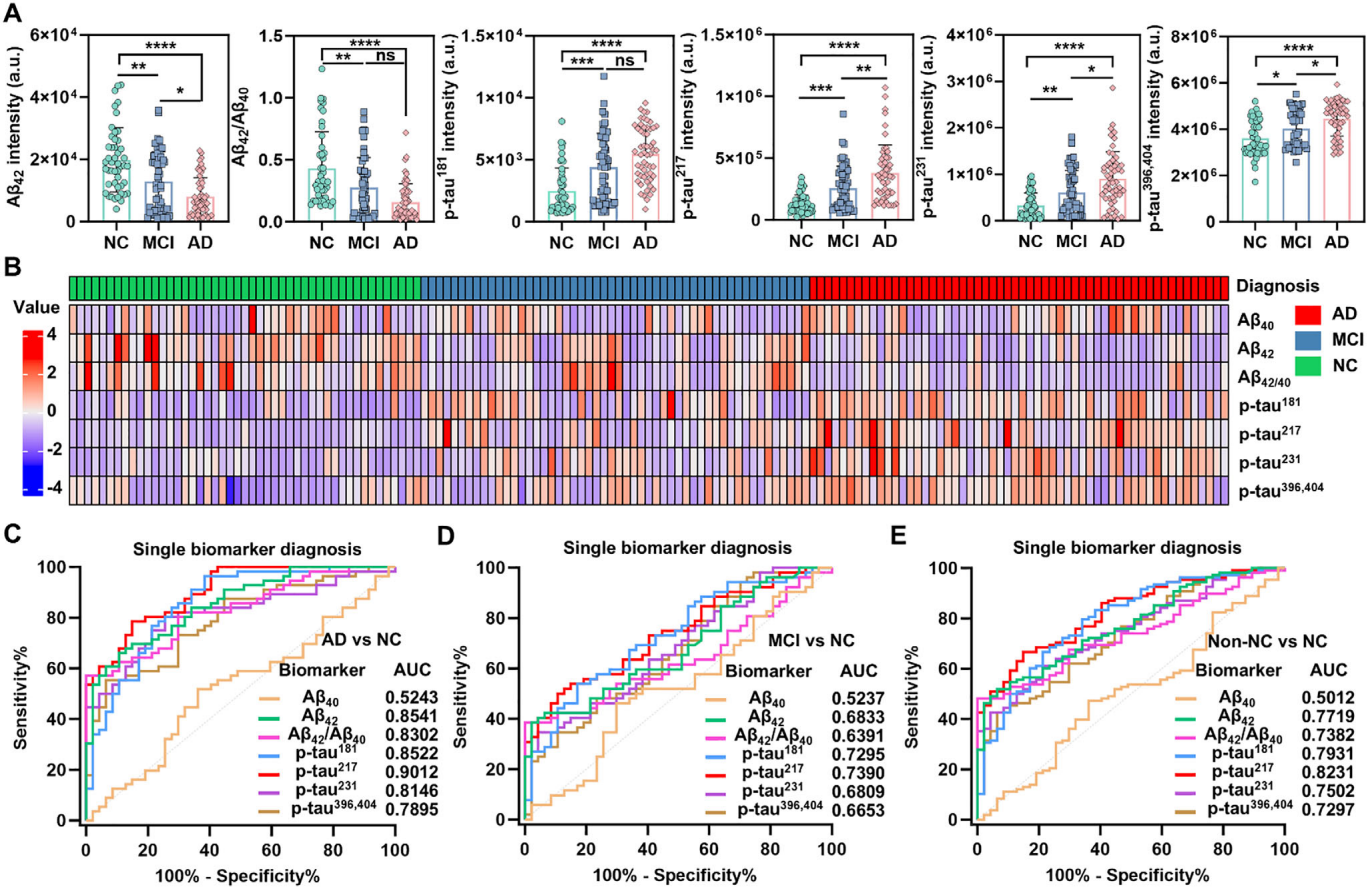
Clinical validation of UCMDA for multi‐biomarker detection. (A) Box plots showing the endpoint fluorescence signals of Aβ_42_, Aβ_42_/Aβ_40_, p‐tau^181^, p‐tau^217^, p‐tau^231^, and p‐tau^396,404^ in the plasma of patients with AD (n = 56), individuals with AD‐related MCI (n = 52), and NCs (n = 47). (B) Heat map analysis of the distribution of Aβ_40_, Aβ_42_, p‐tau^181^, p‐tau^217^, p‐tau^231^, and p‐tau^396,404^ in plasma of AD, AD‐related MCI, and NCs. (C‐E) ROC curves of UCMDA for distinguishing AD (C), AD‐related MCI (D), or non‐NCs (E) from NCs based on single biomarker detection. Data are presented as mean ± s.d., ^*^ *p* ≤ 0.05, ^**^ *p* ≤ 0.01, ^***^ *p* ≤ 0.001, and ^****^ *p* ≤ 0.0001. One‐way ANOVA was used for statistical analysis.

Moreover, we evaluated the performance of UCMDA in multi‐biomarker detection in a cohort of 155 clinical plasma samples, including NCs (n = 47), individuals with AD‐related MCI (n = 52), and patients with AD (n = 56). Detailed demographic characteristics of the subjects are listed in Table S5. Similar to the test results of transgenic mice, UCMDA is capable of simultaneously analyzing multiple biomarkers in clinical samples. Consistent with previous studies [36, 37, 38, 39], the results of UCMDA in the clinical samples showed that plasma Aβ_40_ levels were similar in AD, AD‐related MCI, and NC groups (Figure S14). However, Aβ_42_ levels and the Aβ_42_/Aβ_40_ ratio decreased significantly with the progression of dementia (Figure 6A). In contrast, p‐tau protein levels markedly increased as NCs progressed to AD‐related MCI and AD (Figure 6A). The distribution of the six biomarkers was presented in the form of a heat map (Figure 6B).

### 2.7 Machine Learning‐Based Evaluation of UCMDA in the Early Diagnosis of AD

We further compared the performance of different biomarkers in the diagnosis and staging of AD. Different disease stages were determined based on the physician's clinical diagnosis combined with the diagnostic results of Aβ‐PET, clinical dementia rating (CDR), and Montreal cognitive assessment (MoCA). Compared with other biomarkers, plasma p‐tau^217^ showed higher accuracy and specificity in the diagnosis and staging of AD (Figure 6C–E, Figures S15–S17, and Tables S6–S8). This finding is consistent with the results reported in recent studies [40, 41]. Nevertheless, the accuracy and specificity of plasma p‐tau^217^ for the early diagnosis of AD need to be further improved, especially for MCI, as the area under the receiver operating characteristic curve (AUC) was 0.739. These results suggest that single Aβ or p‐tau biomarker tests (e.g., Aβ_42_, Aβ_42_/Aβ_40_, p‐tau^181^, p‐tau^217^, p‐tau^231^, or p‐tau^396,404^) do not appear to provide a reliable diagnosis in the early stages of AD. Notably, this conclusion was also supported by Simoa's ultrasensitive analysis of Aβ_42_, Aβ_42_/Aβ_40_ ratio, and p‐tau^181^ in some clinical samples. The results showed that the AUC value, sensitivity, specificity, precision, and accuracy based on Simoa were comparable to those of UCMDA (Figures S18–S21 and Tables S9–S11), further demonstrating the reliability and accuracy of UCMDA. Thus, combined detection of multiple AD biomarkers in the plasma has the potential to improve the accuracy of early diagnosis for individuals at risk for AD [42, 43].

**FIGURE 7:**
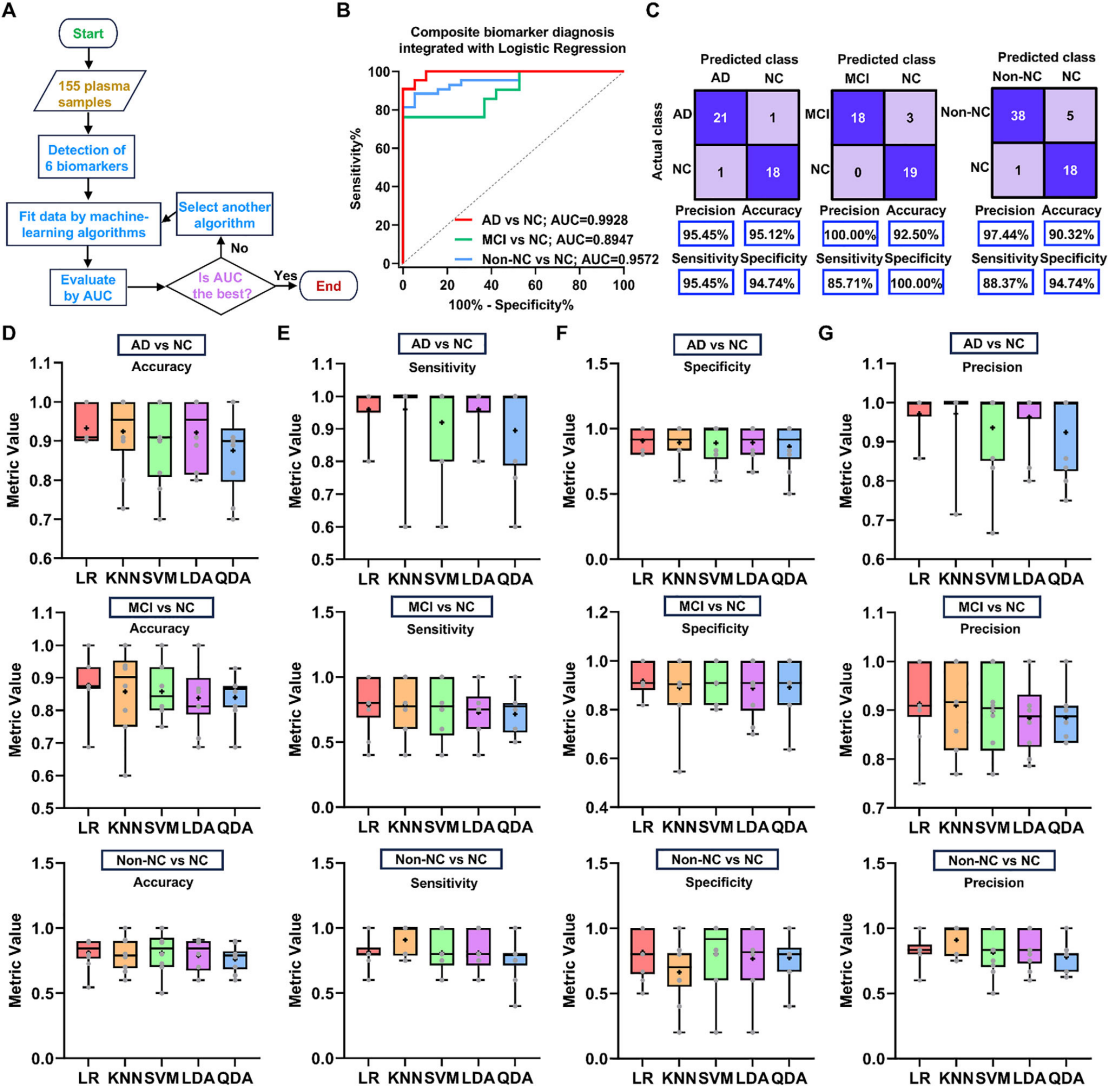
Performance of composite biomarkers in AD diagnosis. (A) Schematic diagram of the screening machine learning algorithm. (B) ROC curves of the LR based on the combination of six biomarkers for distinguishing AD, AD‐related MCI, or non‐NCs from NCs. (C) Precision, accuracy, sensitivity, and specificity of the LR based on the combination of six biomarkers for differentiating AD, AD‐related MCI, or non‐NCs from NCs. (D‐G) The accuracy (D), sensitivity (E), specificity (F), and precision (G) of the different machine learning algorithms based on the combination of six biomarkers for differentiating AD, AD‐related MCI, or non‐NCs from NCs were evaluated with a 10‐fold cross‐validation methodology. Data are presented as mean ± s.d.

Figure 7A presents a schematic representation of an algorithmic screening strategy aimed at enhancing the diagnostic accuracy of AD through the analysis of six plasma biomarkers. Different algorithm analyses showed that LR achieved higher AUC values than support vector machine (SVM, linear), linear discriminant analysis (LDA), k‐nearest neighbors (KNN), and quadratic discriminant analysis (QDA) in distinguishing AD (AUC = 0.9928), AD‐related MCI (AUC = 0.8947), or non‐NCs (AUC = 0.9572) from NCs (Figure 7B, Figure S22 and Tables S12–S14). In addition, the LR algorithm showed superior performance compared with alternative algorithms in distinguishing among AD, AD‐related MCI, non‐NC, and NC, with a precision of ≥ 95.45%, an accuracy of ≥ 90.32%, a sensitivity of ≥ 85.71%, and a specificity of ≥ 94.74% (Figure 7C, Figures S23–S25, and Tables S12–S15). To further confirm the superiority of the LR algorithm, we utilized k‐fold cross‐validation, which has been considered as the gold standard for evaluating algorithm performance [44, 45]. We comprehensively assessed different machine algorithms from four dimensions, including accuracy, sensitivity, specificity, and precision of differentiating AD, AD‐related MCI, non‐NC, and NC. The results showed that the LR significantly outperformed other algorithms on multiple key indicators, and thus was chosen for this study (Figure 7D–G). Specifically, in the most challenging task of distinguishing AD‐related MCI from NC, the LR model demonstrated the highest accuracy (Figure 7D) and, critically, the highest specificity (Figure 7F), which is essential for minimizing false‐positive diagnoses in the context of clinical screening. Although other models like KNN showed high sensitivity, their poor specificity made them unsuitable for practical application. Taken together, these findings suggest that the analysis of multiple AD core biomarker levels using the UCMDA platform combined with the machine learning algorithm could help achieve an accurate diagnosis of AD‐related MCI, with an AUC surpassing 0.89 and an accuracy rate exceeding 92.5%.

## 3 Conclusion

In summary, we established a highly modular and ultrasensitive UCMDA platform for simultaneous detection of multiple AD‐related plasma biomarkers. By integrating composite biomarker analysis with machine learning, this approach substantially improves early diagnostic accuracy using minimally invasive blood testing. Owing to its sensitivity, multiplex capacity, and adaptability through simple substitution of antibody pairs, UCMDA holds considerable promise for broader applications in neurodegenerative disorders, oncology, and cardiovascular diseases.

## 4 Experimental Section

### 4.1 Materials

A TwistAmp Liquid Basic kit was purchased from TwistDx (Cambridge, UK). EnGen Lba Cas12 (Cpf1) was purchased from New England Biolabs. Anti‐Aβ_40_ (A40, cat: V28704) mouse monoclonal antibody (mAb) and anti‐Aβ_40_/Aβ_42_ mouse mAb (6E8, cat: V28702) were ordered from GenScript (Nanjing, China). Pierce Streptavidin Poly‐HRP (cat: 21140) was purchased from ThermoFisher. Anti‐Aβ_42_ mouse mAbs 1F12, 2C6, anti‐tau mouse mAb 1G11, anti‐p‐tau^181^ mouse mAb 1E9, anti‐p‐tau^217^ mouse mAb 1A2, anti‐p‐tau^231^ mouse mAb 2H2, and anti‐p‐tau^396,404^ mouse mAb 4B1 were all produced by our laboratory [46, 47, 48, 49]. Details of antibodies are given in Table S16. Aβ_40_, Aβ_42_, p‐tau^181^, p‐tau^217^, p‐tau^231^, p‐tau^396,404^, tau‐p‐tau^181^, and tau‐p‐tau^396,404^ peptides were custom‐synthesized as lyophilized powders by Royo Biotech Co., Ltd (Shanghai, China) with a purity of >95%, and their sequence information is shown in Table S17. Protein A magnetic nanoparticles (cat: L‐2101A) were obtained from LinkedIn Biotechnology Co., Ltd (Shanghai, China), while carboxyl‐modified magnetic nanoparticles (M1000C) were purchased from Bioeast Biotechnology Co., Ltd (Hangzhou, China). ssDNA, RPA primers, and crRNA were synthesized by Shanghai Sangon Biotechnology Co., Ltd (Shanghai, China), and their sequence information is shown in Tables S1–S3. Human Aβ_40_ (cat: JL41245), Aβ_42_ (cat: JL41255), p‐tau^181^ (cat: JL49331), p‐tau^217^ (cat: JL11325), and p‐tau^231^ (cat: JL47070) ELISA kits were purchased from Shanghai Jianglai Biotechnology Co., Ltd. (Shanghai, China). Horseradish peroxidase (HRP)‐conjugated goat anti‐mouse IgG (Cat: AB_2338504) was provided by Jackson ImmunoResearch Laboratories (Lancaster, USA). High‐sieving agarose (10221ES60), 2×HieffPCR Master Mix (Cat: 10102ES08), nucleic acid gel stains (10204ES76), prestained protein marker (20350ES76), and Super ECL detection kit (36208ES76) were obtained from Shanghai Yeasen Biotechnology Co., Ltd (Shanghai, China).

### 4.2 Dot Blot Assay

Here, 1 µg of p‐tau^181^, p‐tau^217^, p‐tau^231^, and p‐tau^396,404^ were pipetted onto nitrocellulose (NC) membranes. After peptide sample deposition, NC membranes were blocked with 3% BSA for 1 h at 37°C. The membrane was then incubated with anti‐p‐tau^181^, ‐p‐tau^217^, ‐p‐tau^231^, and ‐p‐tau^396,404^ (1:4000) at 37°C for 2 h, followed by incubation with HRP‐conjugated goat anti‐mouse or rabbit IgG (H+L) (1:8000) secondary antibody at 37°C for 1 h. After each step, the membrane was washed three times with TBS‐T. Finally, the immunological signal was detected using ECL substrate on Tanon 5200 Multi (Shanghai, China).

### 4.3 Western Blotting

Here, 5 µg of Aβ_42_ and Aβ_40_ peptides were mixed with SDS‐PAGE sample loading buffer and then electrophoresed on 12% reduced tris‐tricine SDS‐polyacrylamide gels. All proteins were transferred to nitrocellulose membranes at 160 mA for 1 h at 4°C. The membrane was blocked with 3% BSA and then incubated with anti‐Aβ_42_ or Aβ_40_ antibody (1:2000) at 37°C for 2 h. Subsequently, the membrane was incubated with HRP‐conjugated goat anti‐mouse IgG (H+L) (1:8000) at 37°C for 1 h, and the immunological signal was detected using ECL‐substrate on Tanon 5200 Multi.

### 4.4 ELISA

Indirect ELISA was performed to evaluate antibody selectivity. To evaluate antibody selectivity for Aβ_40_ and Aβ_42_, 96‐well plates (NEST Biotechnology Co., Ltd., Wuxi, China) were coated with 0.5 µg of Aβ_40_ or Aβ_42_ peptides per well and then incubated with mouse mAb A40, 6E8, 1F12, and 2C6 (1:3000) at 37°C for 1 h. Subsequently, HRP‐conjugated goat anti‐mouse IgG (H+L) (1:8000) was added and incubated at 37°C for 1 h. Finally, a soluble TMB substrate solution (Abcam, cat: ab171523) was added to detect the immune response, and the absorbance of each well was detected at 450 nm. For evaluating the selectivity of antibodies for different p‐tau proteins, each well of a 96‐well plate was coated with 0.5 µg of p‐tau^181^, p‐tau^217^, p‐tau^231^, or p‐tau^396,404^ peptides, and the antibodies 1E9, 1A2, 2H2, and 4B1 (1:5000) were then added, followed by incubation at 37°C for 1 h. Subsequently, HRP‐conjugated goat anti‐mouse IgG (H+L) or HRP‐conjugated goat anti‐rabbit IgG (H+L) (1:8000) secondary antibody was added, followed by incubation at 37°C for 1 h. Finally, a soluble TMB substrate solution was added to detect the immunoreaction, and the absorbance of each well was measured at 450 nm.

### 4.5 Screening of Preferred Antibody Pairs for Different Aβ and p‐tau Detections

Biotinylated anti‐Aβ and p‐tau antibodies were generated based on the reaction of antibody amino groups with biotin N‐hydroxysuccinimide (NHS) ester. The preferred antibody pair for the detection of different Aβ isoforms and multiple p‐tau proteins was selected based on sandwich ELISA. For Aβ_40_ or Aβ_42_ detection, 96‐well plates were coated with 0.5 µg of anti‐Aβ_40_ mAb (A40) or anti‐Aβ_42_ mAb (1F12) per well as capture antibody and incubated at 37°C for 2 h, followed by blocking with 3% BSA. Subsequently, 100 ng of Aβ_40_ or Aβ_42_ peptides was added to each well for 1 h incubation at 37°C, followed by the addition of biotinylated anti‐Aβ_40_ (6E8) for Aβ_40_ detection or anti‐Aβ_42_ (2C6) for Aβ_42_ detection. For p‐tau^181^, p‐tau^217^, p‐tau^231^, and p‐tau^396,404^ detection, 96‐well plates were coated with 0.5 µg of anti‐tau mouse mAb 1G11 per well as capture antibody and incubated at 37°C for 2 h, followed by blocking with 3% BSA. Subsequently, 50 µL of plasma was collected from 16‐month‐old htau mice and added to each well, followed by 1 h incubation at 37°C. Biotinylated anti‐p‐tau^181^, ‐p‐tau^217^, ‐p‐tau^231^, and ‐p‐tau^396,404^ were then added to detect p‐tau^181^, p‐tau^217^, p‐tau^231^, and p‐tau^396,404^, respectively. Finally, streptavidin‐coupled poly‐HRP was used to visualize the immunoreaction in each well.

### 4.6 Design of ssDNA, RPA Primers, and crRNA

Six ssDNA sequences were constructed, and their secondary structures were evaluated using the mFold web server. The corresponding Gibbs free energy (ΔG) values were determined with OligoAnalyser. RPA primers targeting each ssDNA were generated via the NCBI primer‐BLAST platform, in accordance with the guidelines provided in the TwistAmp assay design manual. Candidate crRNAs for each ssDNA were subsequently screened using CHOPCHOP, based on established design criteria for Cas12a crRNA.

### 4.7 Preparation and Purification of Antibody‐ssDNA Conjugates

Antibody‐ssDNA conjugates were prepared by a chemical coupling reaction between the amide group of the antibody and the sulfhydryl group of ssDNA via the NHS‐dPEG‐Mal linker. The preparation process was carried out as described previously [50]. The ssDNA‐SH reduction reaction was performed in a 100 µL solution containing ssDNA‐SH (25 µL, 1 mM), 1× PBS‐EDTA (55 µL), and DTT (20 µL, 500 mm in 1× PBS‐EDTA). After the completion of the reduction reaction, DTT and EDTA were removed by ultrafiltration using a 1 kDa cutoff centrifugal filter device. During this period, 3.4 µL of NHS‐dPEG‐Mal (0.85 mg/mL) was added to anti‐Aβ or p‐tau antibody (100 µg) for 1.5 h at 4°C, and the excess crosslinker was removed by ultrafiltration using a 50 kDa cutoff centrifugal filter unit. Subsequently, the reduced ssDNA reacted with antibody‐dPEG‐Mal at 4°C on a rotator overnight, with an antibody/ssDNA molar ratio of approximately 1:11. Finally, unconjugated DNA oligonucleotides were removed by ultrafiltration using a 50 kDa cutoff centrifugal filter unit.

### 4.8 Preparation of Immunomagnetic Beads

Antibody‐modified magnetic beads were prepared, wherein the amino group of the antibody and the carboxyl group of the magnetic bead bonded via a chemical reaction. The detailed experimental steps were the same as those reported in previous studies [51].

### 4.9 1‐Plexed and 6‐Plexed RPA Assays

RPA reactions were performed using commercially available kits. For a typical 1‐plexed RPA assay, a 10 µL reaction mixture contained 0.48 µM forward primer and reverse primers, 5 µL of 2× reaction buffer, 1.84 mM dNTP, 1 µL of 10× Basic E‐mix, 0.5 µL of 20× Core reaction mix, 0.5 µL MgOAc, and 1–10 µL DNA template. For the 6‐plexed RPA assay, six forward and reverse primers were mixed to prepare a primer pool, and the concentration of each primer in the primer pool was adjusted to 10 µM. The composition of the 6‐plexed RPA assay was as follows: 0.48 µM each forward and reverse primers, 25 µL of 2× reaction buffer, 9.2 mM dNTPs, 5 µL of 10× Basic E‐mix, 2.5 µL of 20× Core reaction mixture, 2.5 µL of MgOAc, and 10–20 µL of DNA template. The mixture was vortexed briefly and incubated at 37°C for 30 min. For the negative control, the DNA template was replaced with ddH_2_O, with the rest of the steps remaining the same as above.

### 4.10 CRISPR Reaction

The CRISPR reaction was performed in a buffer containing 5–10 µL of RPA product, 0.67 µm crRNA, 0.13 µm Cas 12a, 0.67 µm FAM‐BHQ reporter oligonucleotide, 2 µL of 10× NEB buffer, and 80 U RNase inhibitor [52]. The mixture was incubated at 37°C for 30 min. For fluorescence analysis, the cleavage results were dynamically characterized on the QuantStudio 3 real‐time PCR system, and the fluorescence signal was recorded every 30 s.

### 4.11 Steps for the Detection of Biomarkers via UCMDA

A standard UCMDA mainly includes three steps: immuno‐capture, RPA, and CRISPR reaction. In this study, the buffer sample or plasma sample (50 or 100 µL) was first added to the magnetic beads modified with anti‐Aβ_40_, Aβ_42_, or tau antibodies for biomarker enrichment. Subsequently, antibody‐ssDNA conjugates that specifically recognize Aβ_40_, Aβ_42_, p‐tau^181^, p‐tau^217^, p‐tau^231^, or/and p‐tau^396,404^ were added for single biomarker detection or simultaneous detection of six biomarkers. The formed antibody‐modified magnetic beads@biomarker@antibody‐ssDNA sandwich structure was washed with TBS‐T, and then the bound antibody‐ssDNA was eluted as a DNA template for 1‐plexed or 6‐plexed RPA detection. Finally, the 1‐plexed or 6‐plexed RPA products were used for CRISPR Cas12a cleavage.

### 4.12 Sensitivity and Specificity Evaluation of UCMDA

For sensitivity evaluation, different concentrations (100 pg/mL, 10 pg/mL, 1 pg/mL, 100 fg/mL, 10 fg/mL, and 1 fg/mL) of Aβ_40_, tau‐p‐tau^181^, and tau‐p‐tau^396,404^ dissolved in TBS buffer were prepared for sensitivity testing. In addition, plasma samples of 5×FAD and htau mice were prepared at different dilutions (10^1^, 10^2^, 10^3^, 10^4^, and 10^5^) for Aβ_42_, p‐tau^217^, and p‐tau^231^ sensitivity testing. Further, buffer samples containing the same concentrations of Aβ_40_, Aβ_42_, p‐tau^181^, p‐tau^217^, p‐tau^231^, and p‐tau^396,404^ (1 ng/mL) were prepared for specificity evaluation. UCMDA was then performed on each sample.

### 4.13 Machine Learning Algorithms

Machine learning techniques were performed by R language (version 4.3.1). A dataset was formed using composite biomarker profiles (Aβ_42_, Aβ_40_, p‐tau^181^, p‐tau^217^, p‐tau^231^, and p‐tau^396,404^) of 155 samples to distinguish between AD, AD‐MCI, and NC. The label for each sample in the dataset was their known diagnostic status (AD, AD‐MCI, or NC). The dataset was split into a training set (60%) and a test set (40%) for model training and model testing, respectively. Model training was performed using five machine learning algorithms, including LR, SVM (linear), LDA, KNN, and QDA. Five algorithms were estimated with the default settings of the R function. The performance of each model on the test set was evaluated using sensitivity, specificity, accuracy, and AUC. The model demonstrating the best performance would be chosen for further use.

### 4.14 K‐Fold Cross Validation

To ensure a robust and unbiased evaluation of model performance and to avoid overfitting, we employed a 10‐fold cross‐validation (CV) strategy. The entire dataset was first randomly partitioned into 10 equally sized subsets, or ‘folds’. The CV process was then iterated 10 times. In each iteration, one unique fold was held out as the test set for validation, while the remaining nine folds were combined to form the training set. The model was trained on the training data and subsequently evaluated on the held‐out test fold. This process was repeated until every fold had served as the test set exactly once.

The performance of each model in each fold was assessed using accuracy, sensitivity, specificity, and precision. The final reported performance metrics for each algorithm represent the mean and standard deviation of the values obtained across all 10 folds. This methodology was used to comprehensively compare five distinct algorithms: LR, SVM (Linear), LDA, KNN, and QDA. The analysis was implemented using the caret package in R, which standardizes the model training and evaluation pipeline. The model demonstrating the most consistent and superior performance across all metrics was selected for the final diagnostic evaluation.

### 4.15 Statistics and Reproducibility

No statistical method was used to predetermine sample size. No data were excluded from the analyses. Data are expressed as mean value or mean value ± standard deviation (s.d.). Statistical analysis was performed using GraphPad Prism 8.0 software. Student's t‐test was used for two‐group comparisons. One/two‐way analysis of variance was used to compare multiple groups. The significance cut‐off was set at ^*^ *p* ≤ 0.05, ^**^ *p* ≤ 0.01, ^***^ *p* ≤ 0.001, and ^****^ *p* ≤ 0.0001.

### 4.16 Ethical Approval Statements

All animal procedures were reviewed and approved by the Institutional Animal Care and Use Committee of Hainan University (HNUAUCC‐2025‐00428). The use of human plasma samples was approved by the Ethics Committee of Xuanwu Hospital, Capital Medical University (ClinicalTrials.gov ID: NCT04696315). All experiments were conducted in accordance with relevant guidelines and regulations. Informed consent was obtained from all participants.

## Author Contributions

**Xuewei Du**: formal analysis, validation. **Shuai Ding**: investigation, validation. **Can Sheng**: resources. **Ming Wang**: resources. **Haiming Luo**: supervision, conceptualization, resources, funding acquisition, writing – review and editing, project administration. **Yaoqiang Shi**: resources. **Changwen Yang**: methodology, software, data curation, formal analysis, visualization, validation. **Qian Yao**: investigation. **Liding Zhang**: investigation, validation, formal analysis, writing – original draft, methodology. **Ying Han**: resources.

## Conflicts of Interest

The authors declare no conflicts of interest.

## Supporting information




**Supporting File**: advs75983‐sup‐0001‐SuppMat.pdf.

## Data Availability

The data that support the findings of this study are available from the corresponding author upon reasonable request.
