# Supplementary material for: Machine Learning‐Enhanced Ultrasensitive Immuno‐CRISPR Array Facilitates Early Diagnosis of Alzheimer's Disease by Detecting Multiple Plasma Biomarkers
Source: Adv Sci (Weinh). 2026 Jun 9:e75983. Online ahead of print. doi: 10.1002/advs.75983 (PMC13336432; doi:10.1002/advs.75983)
Supplement: Supplementary file 1 — Supporting File: advs75983‐sup‐0001‐SuppMat.pdf. [file ADVS-9999-e75983-s001.pdf]

Supporting Information

Machine learning-enhanced ultrasensitive immuno-CRISPR array facilitates early diagnosis of Alzheimer's disease by detecting multiple plasma biomarkers

*Liding Zhang[1†], Changwen Yang[2†], Qian Yao[3], Xuewei Du[1], Shuai Ding[1], Yaoqiang Shi[4], Can Sheng[5], Ming Wang[3], Ying Han[1,6], Haiming Luo[1,2]**

[1]Key Laboratory of Biomedical Engineering of Hainan Province, School of Biomedical Engineering, Hainan University, Haikou 570228, China

[2]MOE Key Laboratory for Biomedical Photonics, Wuhan National Laboratory for Optoelectronics, Huazhong University of Science and Technology, Wuhan 430070, China

[3]Department of Clinical Laboratory, Renmin Hospital of Wuhan University, Wuhan 430060, China

[4]Institute of Basic and Clinical Medicine, The First People's Hospital of Yunnan, Kunming 650032, China

[5]Department of Neurology, the Affiliated Hospital of Jining Medical University, Jining 272000, China

[6]Department of Neurology, Xuanwu Hospital of Capital Medical University, Beijing 100053, China

*Corresponding author: Haiming Luo, hemluo@hainanu.edu.cn;

Key Laboratory of Biomedical Engineering of Hainan Province, School of Biomedical Engineering, Hainan University, Haikou 570228, China;

Liding Zhang and Changwen Yang contributed equally to this work.

Table of Contents

Figure S1. Characterization of anti-Aβ conformation-dependent antibodies.
Figure S2. Characterization of phosphorylated tau selective antibodies.
Figure S3. Sequence specificity analysis between different ssDNA.
Figure S4. Selectivity of RPA primers.
Figure S5. Optimization of crRNA scaffold sequence.
Figure S6. Evaluation of crRNA selectivity.
Figure S7. Synthesis and characterization of antibody-ssDNA.
Figure S8. Evaluation of the biological activity of antibody-ssDNA.
Figure S9. Specificity of UCMDA.
Figure S10. Sensitivity of UCMDA in standard protein solutions.
Figure S11. Sensitivity of UCMDA in the plasma of transgenic mice.
Figure S12. Kinetic curves of UCMDA for detecting different Aβ and p-tau.
Figure S13. Performance evaluation of UCMDA for detecting six AD biomarkers in transgenic mouse plasma.
Figure S14. The fluorescence intensity of $A\beta_{40}$ in clinical samples.
Figure S15. Performance of UCMDA using the single biomarker in distinguishing AD from NCs.
Figure S16. Performance of UCMDA using the single biomarker in distinguishing AD-related MCI from NCs.
Figure S17. Performance of UCMDA using a single biomarker in distinguishing non-NCs from NCs.
Figure S18. ROC curves of UCMDA and Simoa for distinguishing AD, AD-related MCI, or non-NCs from NCs using the predictor of $A\beta_{42}$, $A\beta_{42}/A\beta_{40}$, or p-tau^{181}.
Figure S19. Comparison of the performance of UCMDA and Simoa in distinguishing AD from NCs in the same clinical samples.
Figure S20. Comparison of the performance of UCMDA and Simoa for distinguishing AD-related MCI from NCs in the same clinical samples.
Figure S21. Comparison of the performance of UCMDA and Simoa in distinguishing non-NCs from NCs in the same clinical samples.
Figure S22. ROC curves of different algorithms for distinguishing AD, MCI, or non-NCs from NCs.
Figure S23. Comparison of the performance of different algorithms in distinguishing AD from NCs.
Figure S24. Comparison of the performance of different algorithms in distinguishing AD-related MCI from NCs.
Figure S25. Comparison of the performance of different algorithms in distinguishing non-NCs from NCs.
Table S1. ssDNA sequences used in UCMDA
Table S2. RPA primer sequences used in UCMDA
Table S3. crRNA sequences and the probe used in UCMDA
Table S4. Comparison of UCMDA with other detection methods
Table S5. Demographic characteristics of the participants

Table S6. AUC value, sensitivity, specificity, precision, and accuracy of ROC analysis based on UCMDA using the predictor of single biomarker in distinguishing between AD patients and NCs
Table S7. AUC value, sensitivity, specificity, precision, and accuracy of ROC analysis based on UCMDA using the predictor of the single biomarker in distinguishing between AD-related MCI and NCs
Table S8. AUC value, sensitivity, specificity, precision, and accuracy of ROC analysis based on UCMDA using the predictor of single biomarker in distinguishing between non-NCs and NCs
Table S9. Comparison of AUC value, sensitivity, specificity, precision, and accuracy of UCMDA and Simoa using the predictor of $A\beta_{42}$, $A\beta_{42}/A\beta_{40}$, or p-tau^{181} in distinguishing between AD patients and NCs
Table S10. Comparison of AUC value, sensitivity, specificity, precision, and accuracy of UCMDA and Simoa using the predictor of $A\beta_{42}$, $A\beta_{42}/A\beta_{40}$, or p-tau^{181} in distinguishing between AD-related MCI and NCs
Table S11. Comparison of AUC value, sensitivity, specificity, precision, and accuracy of UCMDA and Simoa using the predictor of $A\beta_{42}$, $A\beta_{42}/A\beta_{40}$, or p-tau^{181} in distinguishing between non-NCs and NCs
Table S12. The AUC value, sensitivity, specificity, precision, and accuracy of different algorithms using the predictor of composite biomarkers detected by UCMDA in distinguishing between AD patients and NCs
Table S13. The AUC value, sensitivity, specificity, precision, and accuracy of different algorithms using the predictor of composite biomarkers detected by UCMDA in distinguishing between AD-related MCI and NCs
Table S14. The AUC value, sensitivity, specificity, precision, and accuracy of different algorithms using the predictor of composite biomarkers detected by UCMDA in distinguishing between non-NCs and NCs
Table S15. The weight of each biomarker in the composite signature of LR

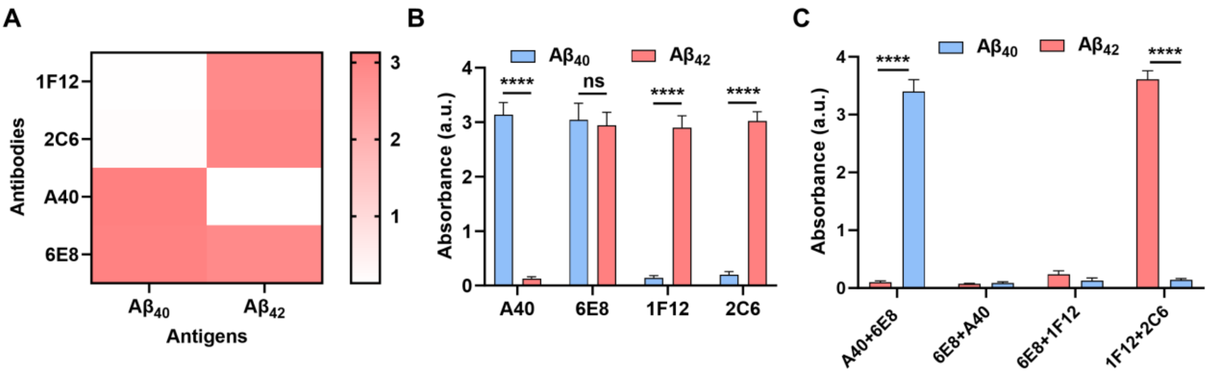

Figure S1 Characterization of anti-Aβ conformation-dependent antibodies. (A, B) Heat map (A) and quantitative analysis (B) of the selectivity of different antibodies for $A\beta_{42}$ and $A\beta_{40}$. (C) Screening of the best antibody pairs for detecting $A\beta_{42}$ and $A\beta_{40}$. Data are presented as mean or mean ± s.d. (n = 4, ****$p \leq 0.0001$).

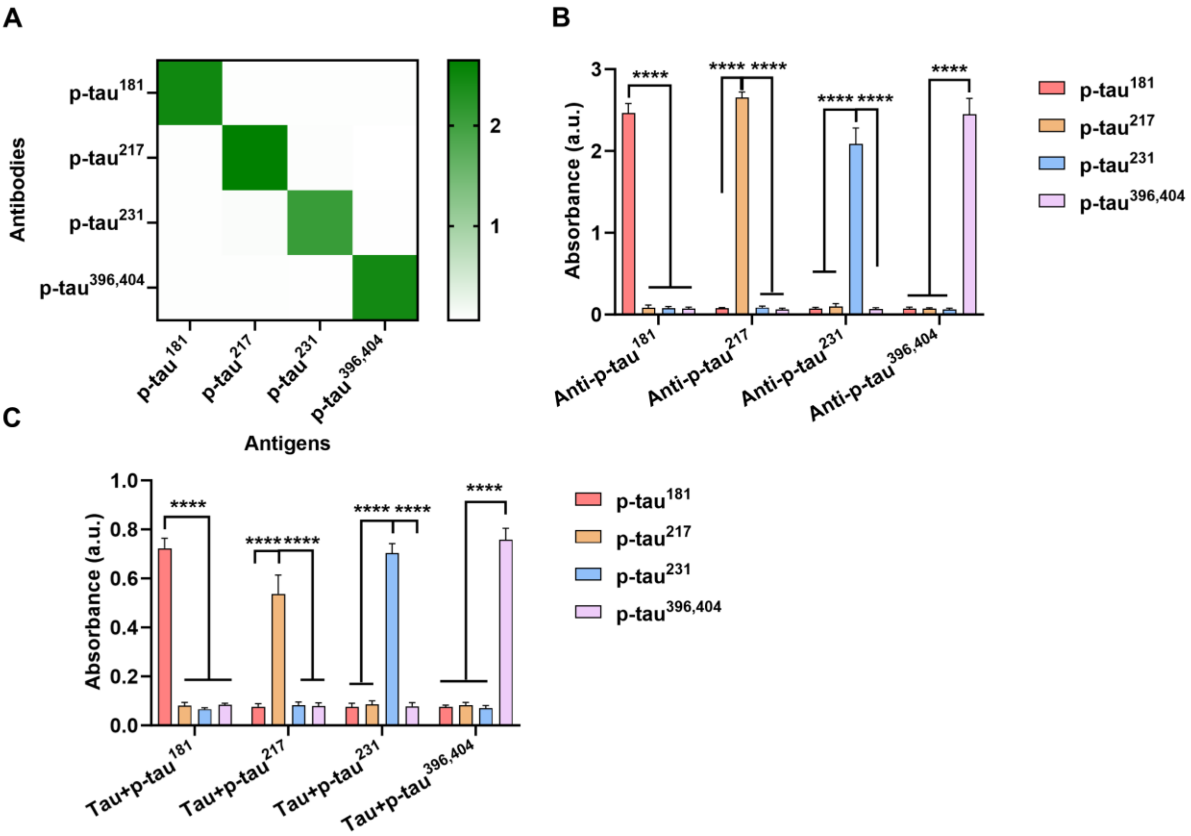

Figure S2 Characterization of phosphorylated tau selective antibodies. (A, B) Heat map (A) and quantitative analysis (B) of antibody selectivity for p-tau^{181}, p-tau^{217}, p-tau^{231}, and p-tau^{396,404}. (C) Specificity of antibody pairs for detecting p-tau^{181}, p-tau^{217}, p-tau^{231}, and p-tau^{396,404}. Data are presented as mean or mean ± s.d. (n = 4, ****$p \leq 0.0001$).

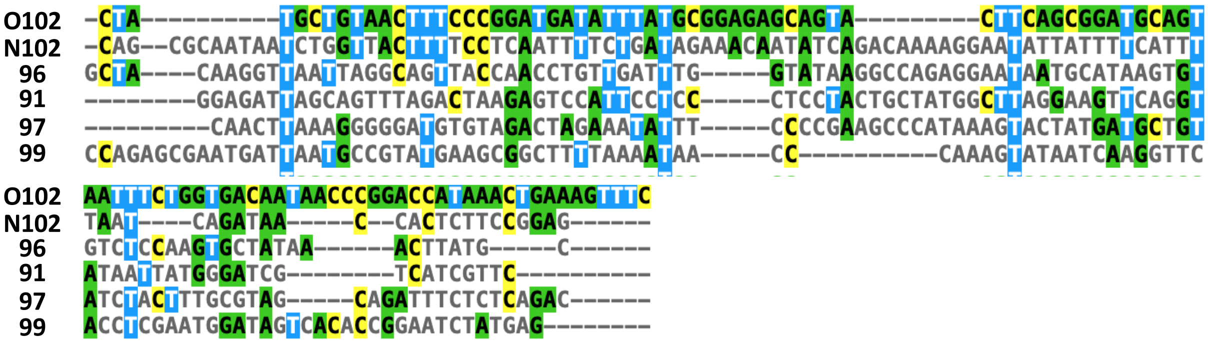

Figure S3 Sequence specificity analysis between different ssDNA.

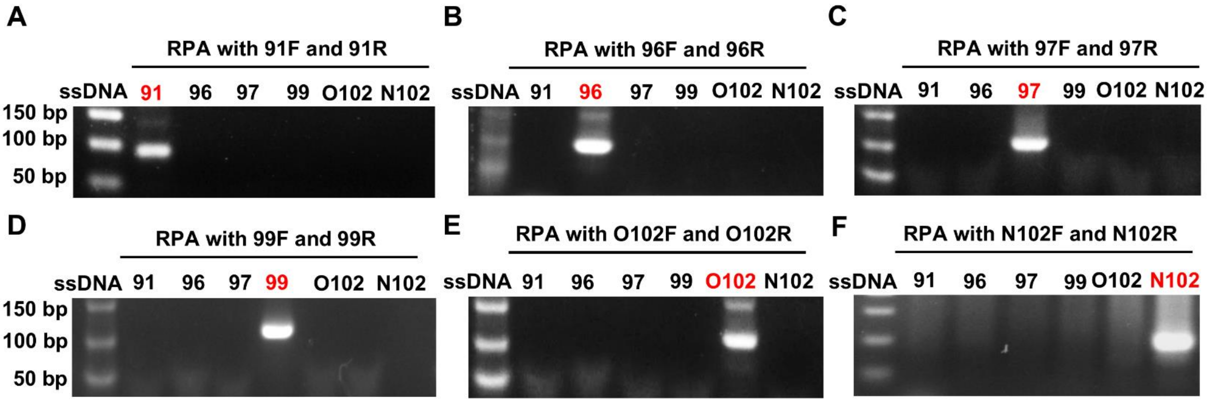

Figure S4 Selectivity of RPA primers. (A-F) Agarose gel images of RPA products for evaluating the selectivity of RPA primers for ssDNA 91 (A), ssDNA 96 (B), ssDNA 97 (C), ssDNA 99 (D), ssDNA O102 (E), or ssDNA N102 (F) in the presence of six ssDNA, including ssDNA 91, ssDNA 96, ssDNA 97, ssDNA 99, ssDNA O102, and ssDNA N102.

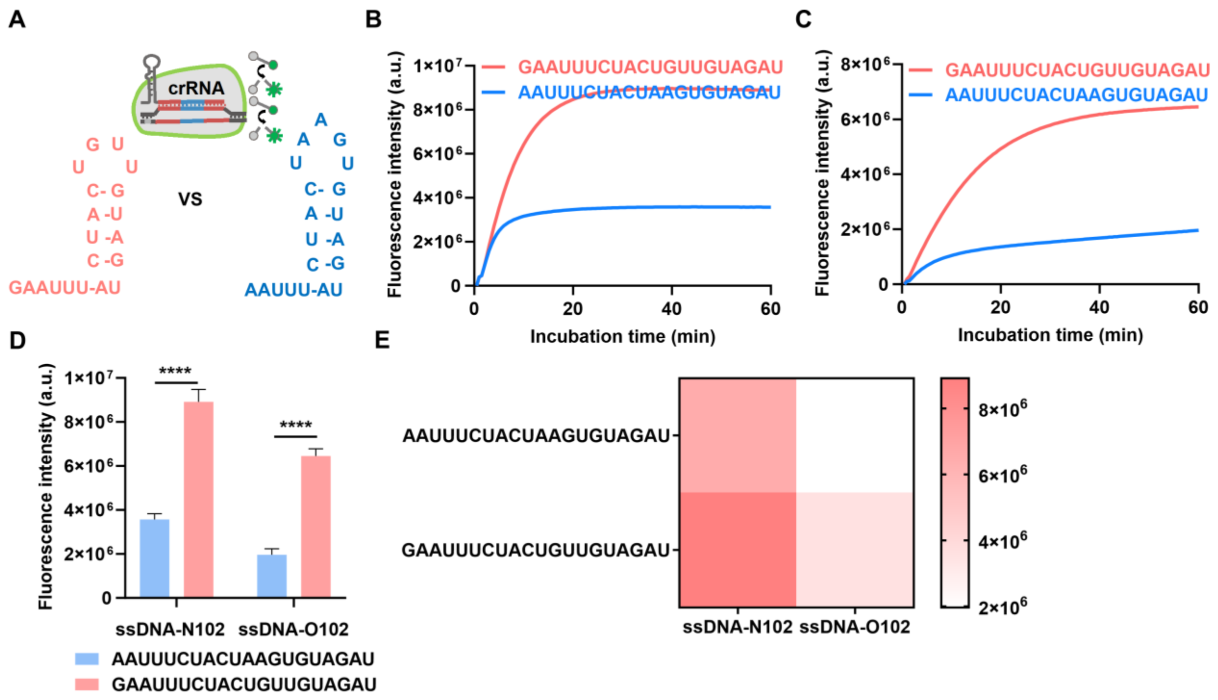

Figure S5 Optimization of crRNA scaffold sequence. (A) Scaffold sequences of two crRNAs. (B, C) Kinetic curves of CRISPR-Cas12a for the detection of dsDNA-O102 and dsDNA-N102 in the presence of crRNA (GAAUUUCUACUGUUGUAGAU) (B) or crRNA (AAUUUCUACUAAGUGUAGAU) (C). (D, E) Quantification (D) and heat map (E) for the analysis of the difference in fluorescence intensity between the two crRNAs. Data are presented as mean or mean ± s.d. (n = 3, ****$p \leq 0.0001$).

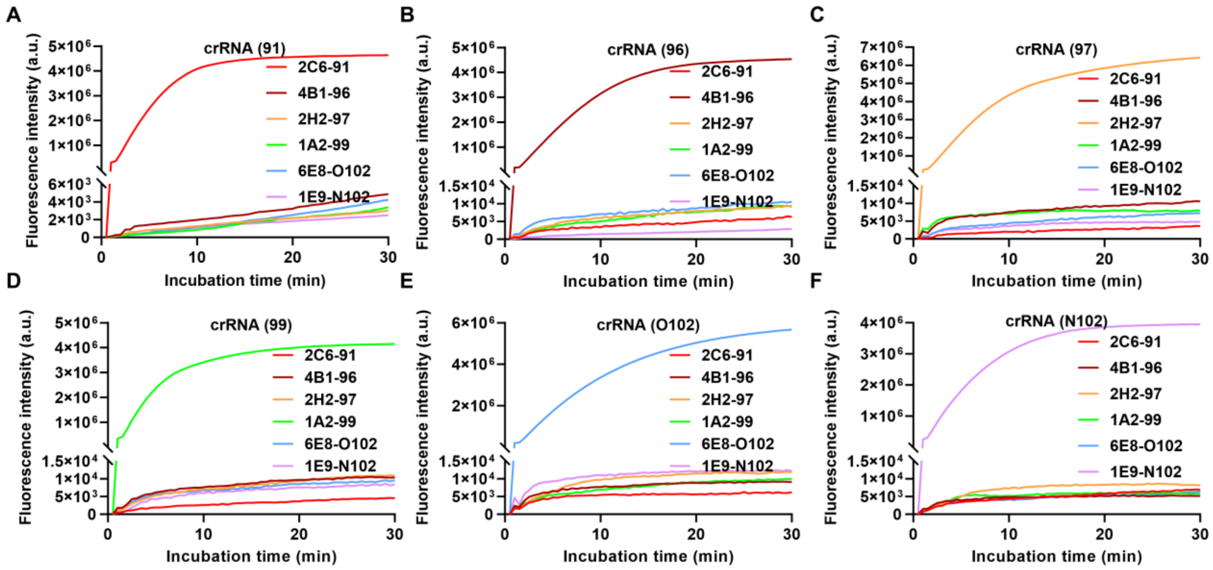

Figure S6 Evaluation of crRNA selectivity. (A-F) Kinetic curves of crRNA 91 (A), crRNA 96 (B), crRNA 97 (C), crRNA 99 (D), crRNA O102 (E), and crRNA N102 (F) for the detection of six different RPA products in the presence of 2C6-ssDNA 91, 4B1-ssDNA 96, 2H2-ssDNA 97, 1A2-ssDNA 99, 6E8-ssDNA O102, or 1E9-ssDNA N102. Data are presented as mean (n = 3).

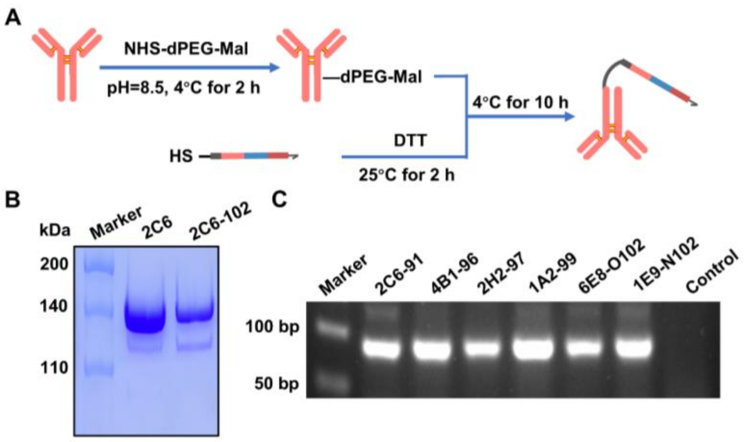

Figure S7 Synthesis and characterization of antibody-ssDNA. (A) Flow chart of antibody-ssDNA synthesis. (B) SDS-PAGE gel image of antibody-ssDNA. (C) Agarose gel images of PCR products using 2C6-ssDNA 91, 4B1-ssDNA 96, 2H2-ssDNA 97, 1A2-ssDNA 99, 6E8-ssDNA O102, and 1E9-ssDNA N102 as templates, respectively.

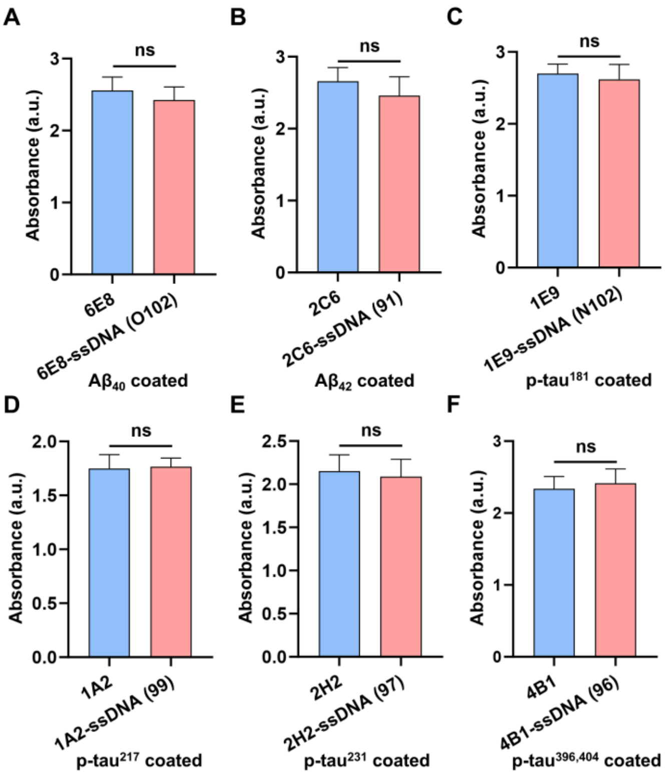

Figure S8 Evaluation of the biological activity of antibody-ssDNA. (A-F) ELISA analysis of the biological activities of 2C6-ssDNA 91, 4B1-ssDNA 96, 2H2-ssDNA 97, 1A2-ssDNA 99, 6E8-ssDNA O102, and 1E9-ssDNA N102 against $A\beta_{40}$ (A), $A\beta_{42}$ (B), p-tau^{181} (C), p-tau^{217} (D), p-tau^{231} (E), and p-tau^{396,404} (F). Data are presented as mean ± s.d. (n = 3).

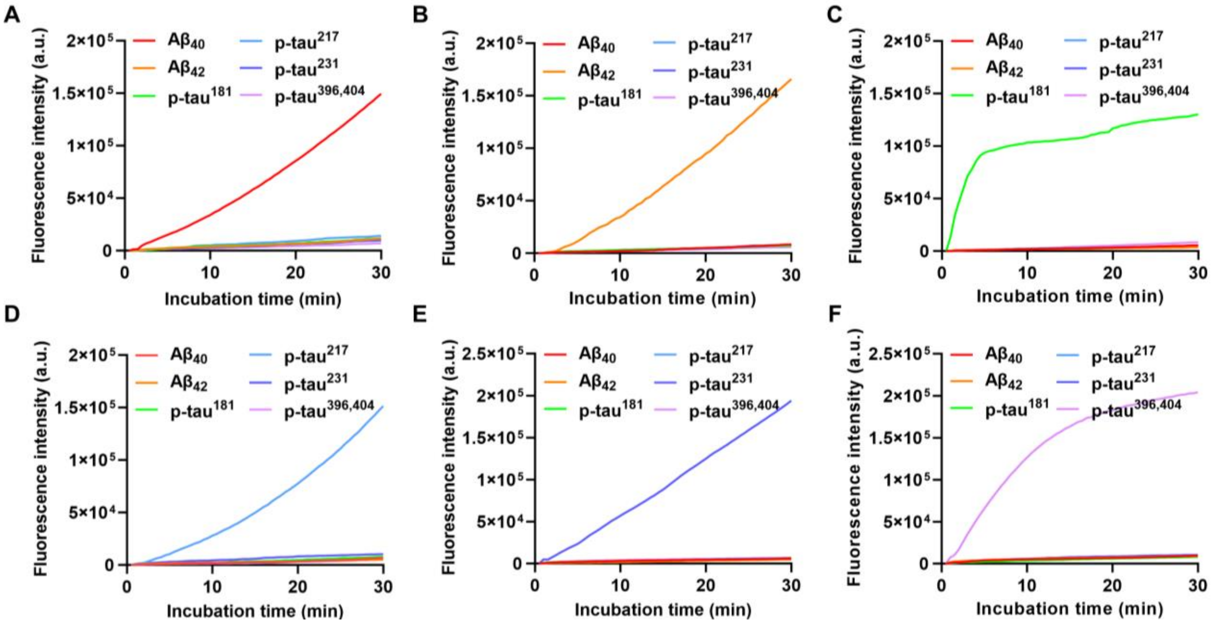

Figure S9 Specificity of UCMDA. (A-F) Kinetic curves of UCMDA for the detection of $A\beta_{40}$ (A), $A\beta_{42}$ (B), p-tau^{181} (C), p-tau^{217} (D), p-tau^{231} (E), and p-tau^{396,404} (F). Data are presented as mean (n = 3).

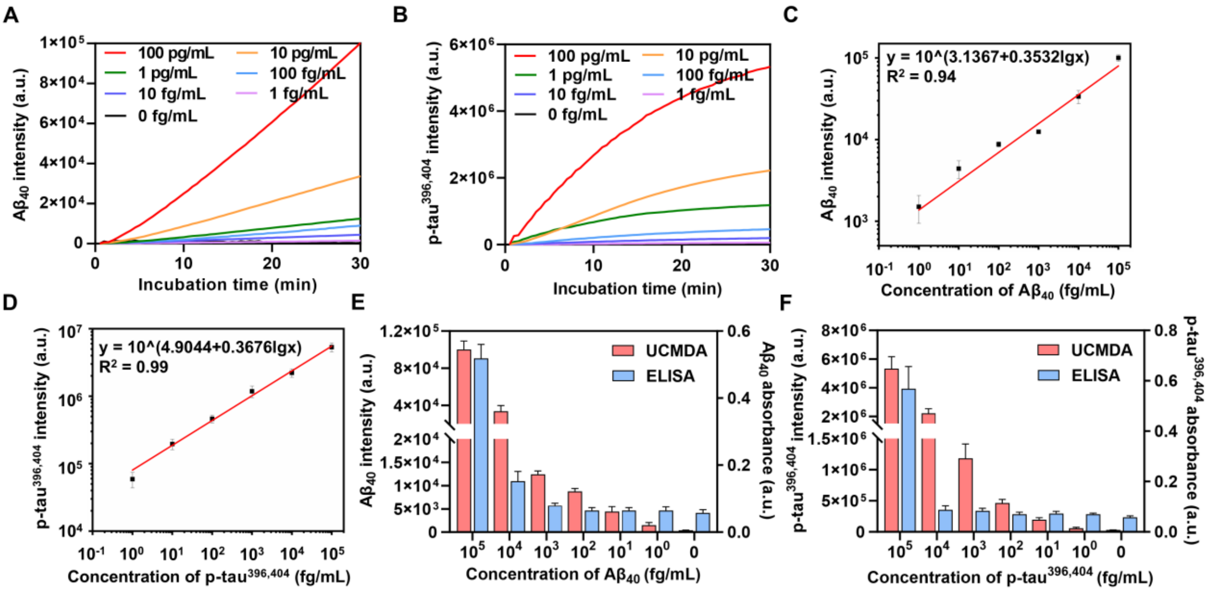

Figure S10 Sensitivity of UCMDA in standard protein solutions. (A, B) Kinetic curves of UCMDA for detecting different concentrations of Aβ_{40} (A) and p-tau^{396,404} (B) diluted in TBS buffer. (C, D) Calibration curves of UCMDA for detecting Aβ_{40} (C) and p-tau^{396,404} (D) in the range of 1 fg/mL to 100 pg/mL. (E, F) Signals of UCMDA and ELISA for detecting different concentrations of Aβ_{40} (E) and p-tau^{396,404} (F). Data are presented as mean or mean ± s.d. (n = 3).

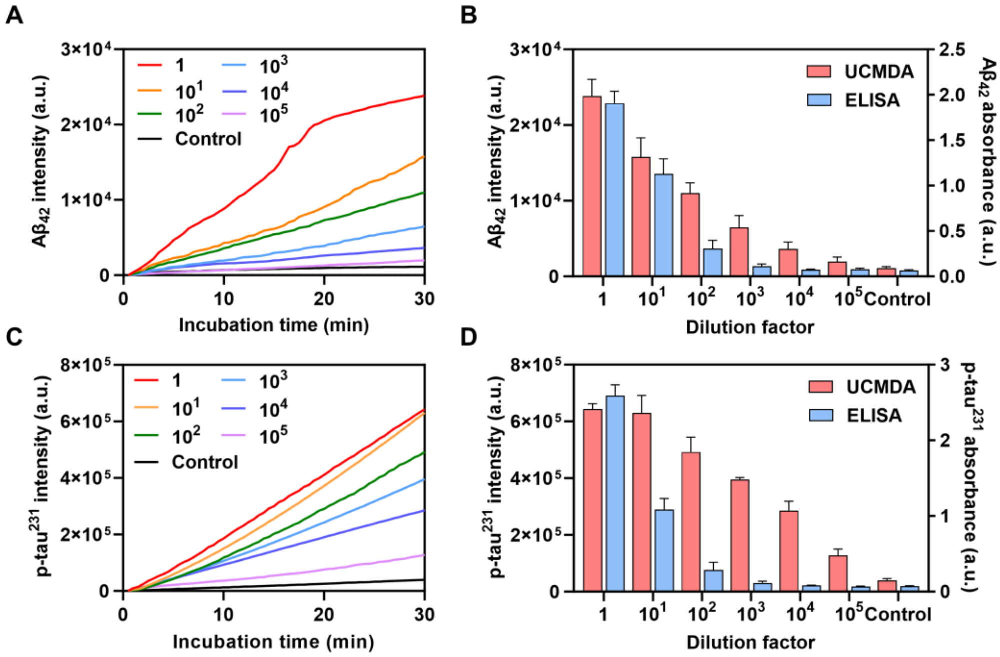

Figure S11 Sensitivity of UCMDA in the plasma of transgenic mice. (A, C) Kinetic curves of UCMDA for detecting Aβ_{42} (A) in 5×FAD and p-tau^{231} (C) in htau mice plasma at dilutions ranging from 1 to 100,000. (B, D) Signals of Aβ_{42} (B) and p-tau^{231} (D) detected by UCMDA and ELISA at different dilution ratios. Data are presented as mean or mean ± s.d. (n = 3).

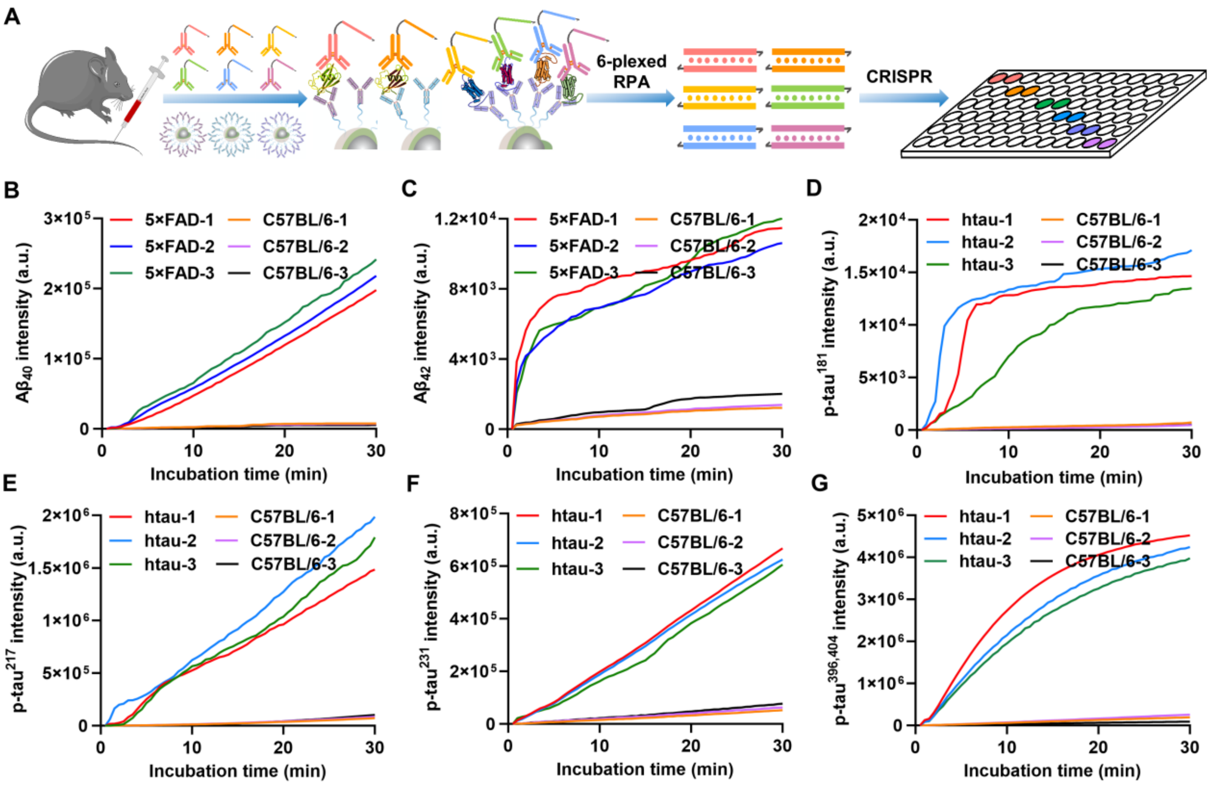

Figure S12 Kinetic curves of UCMDA for detecting different Aβ and p-tau. (A) Schematic illustration of the UCMDA-based plasma detection process. (B-G) Kinetic curves of UCMDA for the detection of $A\beta_{40}$ (B), $A\beta_{42}$ (C), p-tau^{181} (D), p-tau^{217} (E), p-tau^{231} (F), and p-tau^{396,404} (G) in plasma samples of AD transgenic mice or C57BL/6 mice.

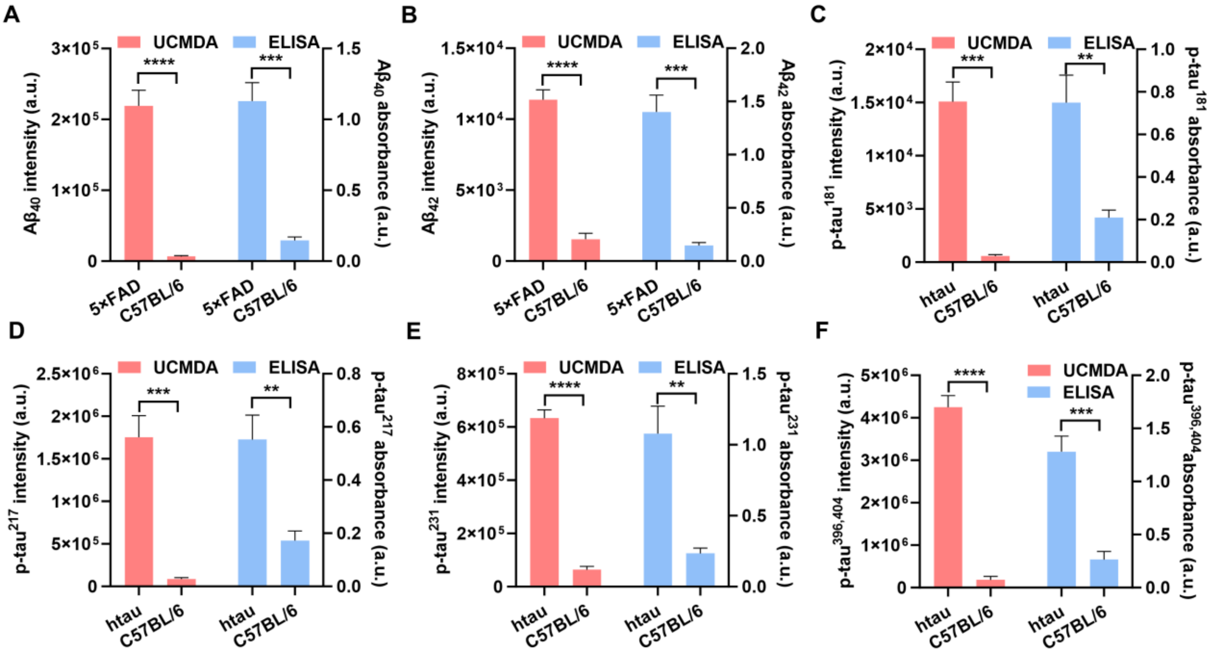

Figure S13 Performance evaluation of UCMDA for detecting six AD biomarkers in transgenic mouse plasma. (A-F) Detection of Aβ_{40} (A), Aβ_{42} (B), p-tau^{181} (C), p-tau^{217} (D), p-tau^{231} (E), and p-tau^{396,404} (F) in mouse plasma samples using UCMDA and standard ELISA. Data are presented as mean ± s.d. (n = 3, $^{**}p \leq 0.01$, $^{***}p \leq 0.001$, and $^{****}p \leq 0.0001$).

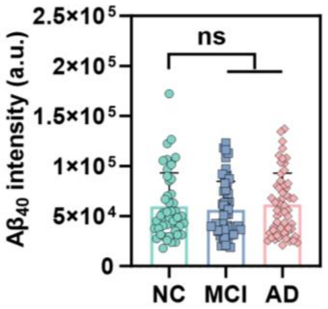

Figure S14 The fluorescence intensity of $A\beta_{40}$ in clinical samples. Box plots showing the endpoint fluorescence intensity of $A\beta_{40}$ in plasma of patients with AD (n = 56), individuals with AD-related MCI (n = 52), and NCs (n = 47). Data are presented as mean ± s.d.

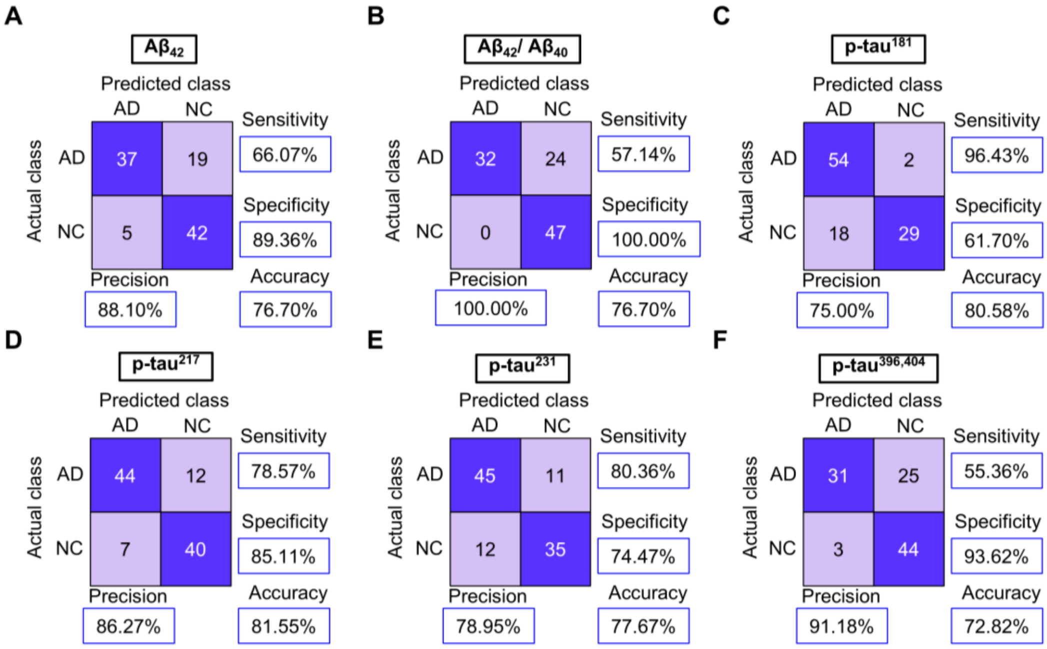

Figure S15 Performance of UCMDA using the single biomarker in distinguishing AD from NCs. (A-F) Sensitivity, specificity, precision, and accuracy of ROC analysis using the predictor of $A\beta_{42}$ (A), $A\beta_{42}/A\beta_{40}$ ratio (B), p-tau^{181} (C), p-tau^{217} (D), p-tau^{231} (E), or p-tau^{396,404} (F) detected by UCMDA in distinguishing patients with AD (n = 56) from NCs (n = 47).

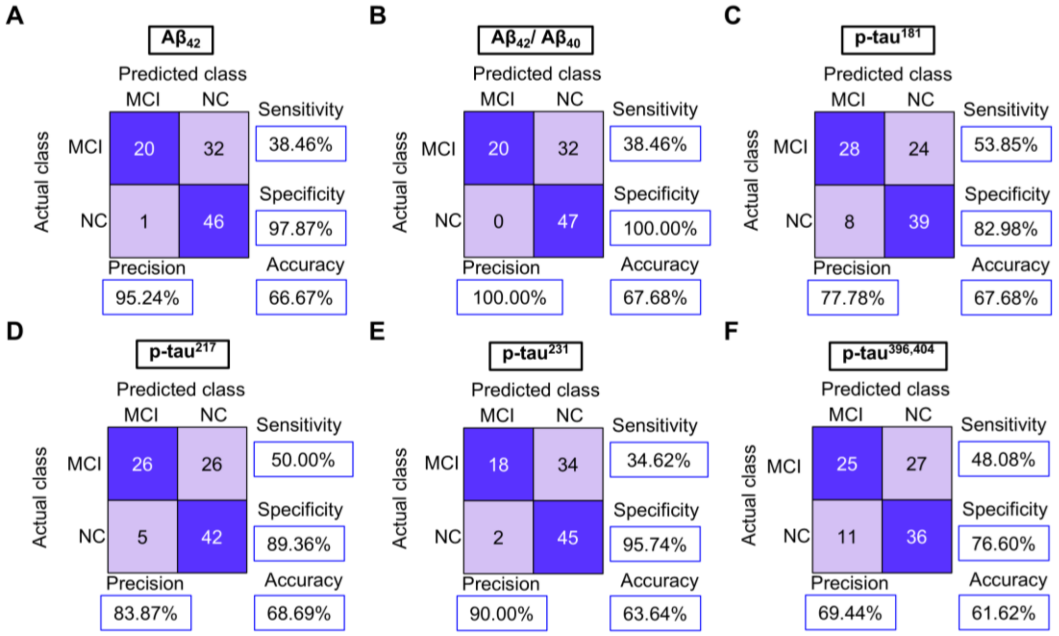

Figure S16 Performance of UCMDA using the single biomarker in distinguishing AD-related MCI from NCs. (A-F) Sensitivity, specificity, precision, and accuracy of ROC analysis using the predictor of $A\beta_{42}$ (A), $A\beta_{42}/A\beta_{40}$ ratio (B), p-tau^{181} (C), p-tau^{217} (D), p-tau^{231} (E), or p-tau^{396,404} (F) detected by UCMDA in distinguishing individuals with AD-related MCI (n = 52) from NCs (n = 47).

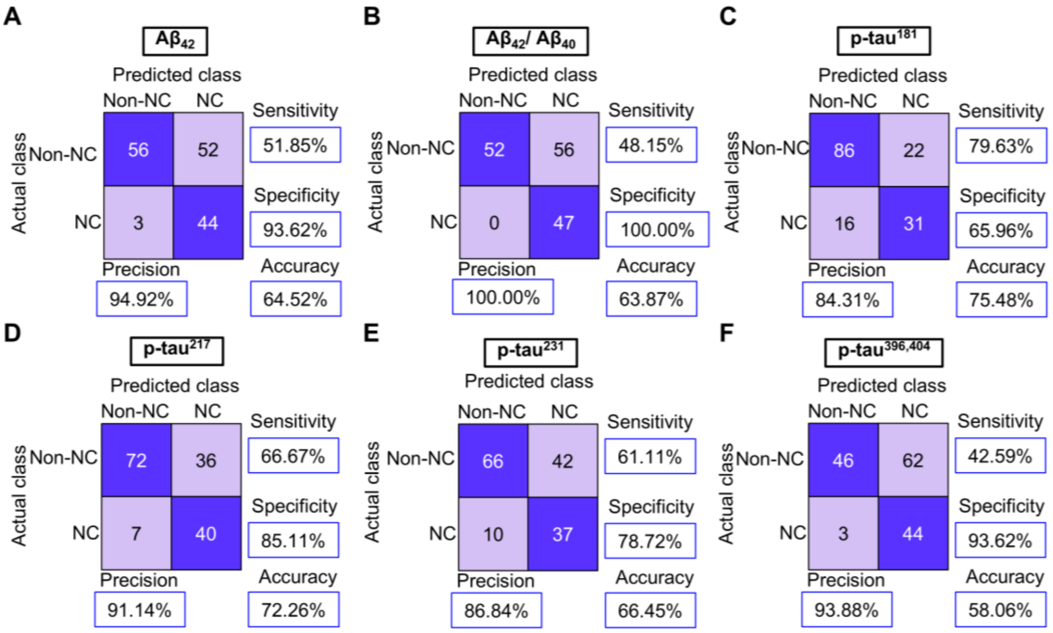

Figure S17 Performance of UCMDA using a single biomarker in distinguishing non-NCs from NCs. (A-F) Sensitivity, specificity, precision, and accuracy of ROC analysis using the predictor of $A\beta_{42}$ (A), $A\beta_{42}/A\beta_{40}$ ratio (B), p-tau^{181} (C), p-tau^{217} (D), p-tau^{231} (E), or p-tau^{396,404} (F) detected by UCMDA in distinguishing non-NCs (n = 108) from NCs (n = 47).

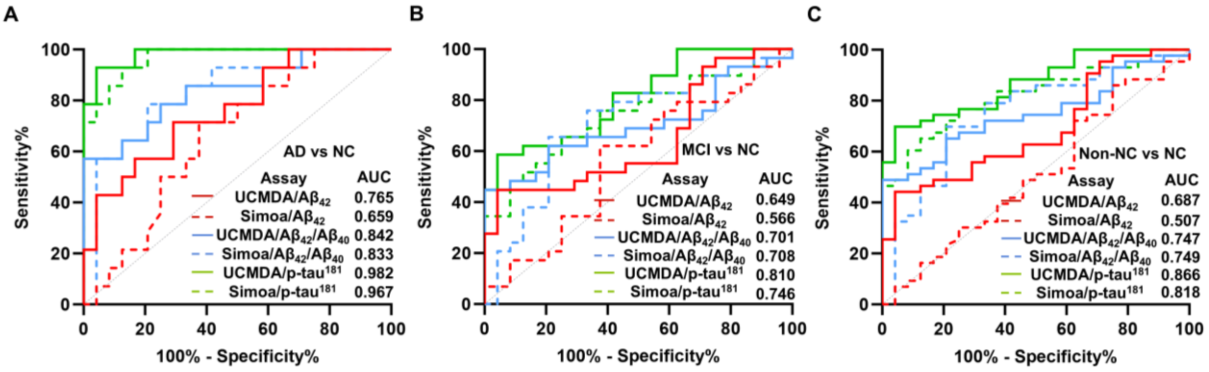

Figure S18 ROC curves of UCMDA and Simoa for distinguishing AD, AD-related MCI, or non-NCs from NCs using the predictor of $A\beta_{42}$, $A\beta_{42}/A\beta_{40}$, or p-tau^{181}. (A-C) ROC curves of UCMDA and Simoa for distinguishing AD (A), AD-related MCI (B), and non-NCs (C) from NCs using the predictor of $A\beta_{42}$, $A\beta_{42}/A\beta_{40}$, or p-tau^{181}. (n = 14 for patients with AD, n = 29 for individuals with AD-related MCI, n = 24 for NCs, and n = 43 for non-NCs.)

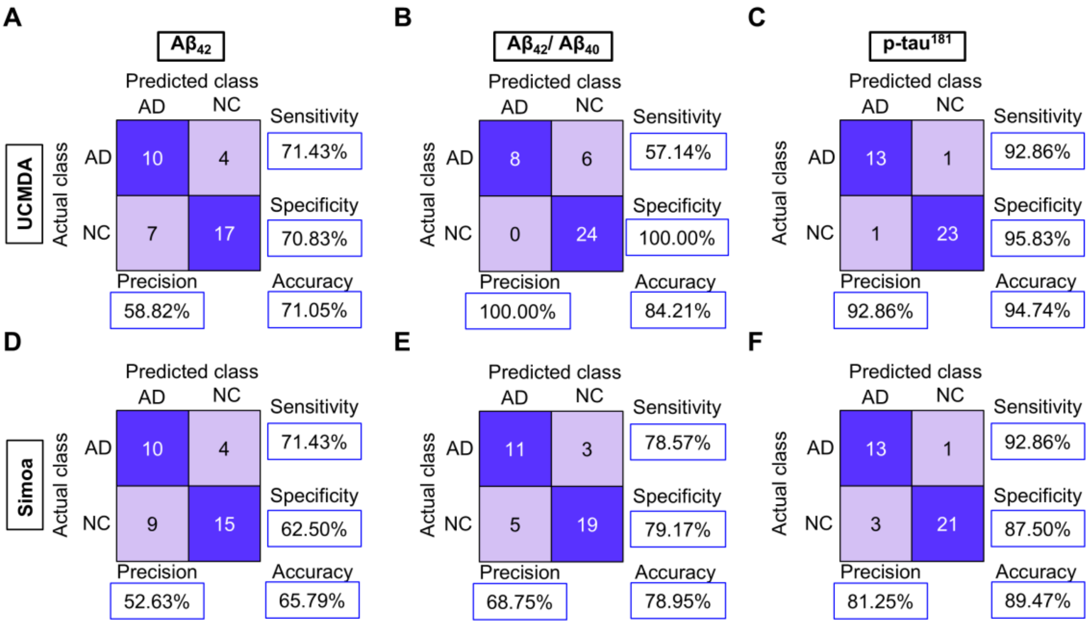

Figure S19 Comparison of the performance of UCMDA and Simoa in distinguishing AD from NCs in the same clinical samples. (A-F) Comparison of the sensitivity, specificity, precision, and accuracy of UCMDA and Simoa in distinguishing AD (n = 14) from NCs (n = 24) using the predictor of $A\beta_{42}$ (A, D), $A\beta_{42}/A\beta_{40}$ ratio (B, E), or p-tau^{181} (C, F).

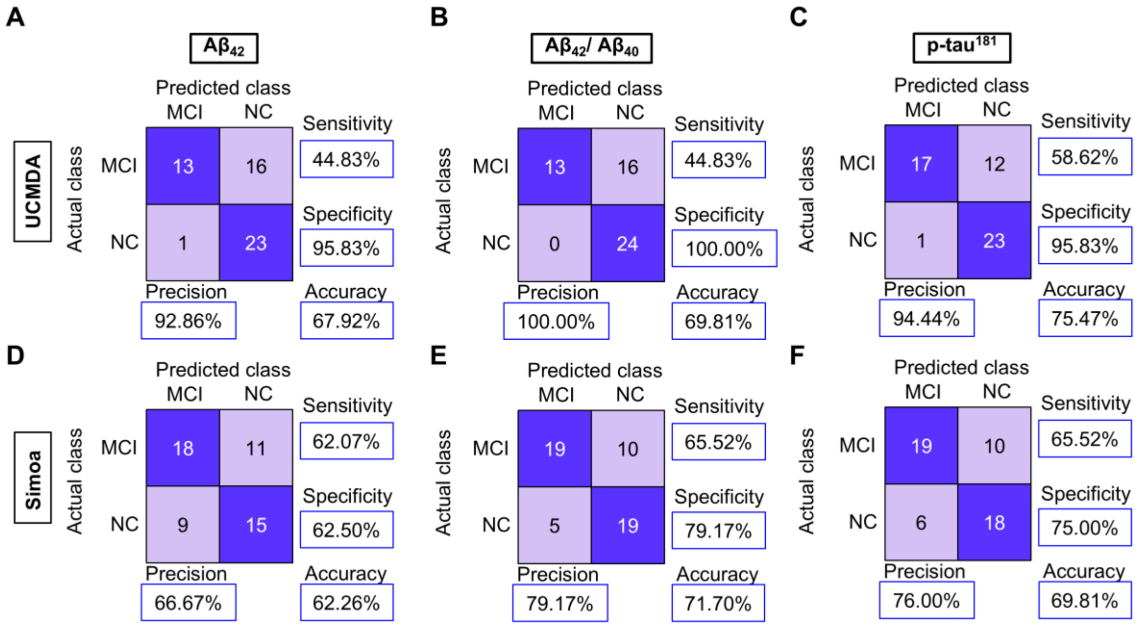

Figure S20 Comparison of the performance of UCMDA and Simoa for distinguishing AD-related MCI from NCs in the same clinical samples. (A-F) Comparison of the sensitivity, specificity, precision, and accuracy of UCMDA and Simoa in distinguishing AD-related MCI (n = 29) from NCs (n = 24) using the predictor of $A\beta_{42}$ (A, D), $A\beta_{42}/A\beta_{40}$ ratio (B, E), or p-tau^{181} (C, F).

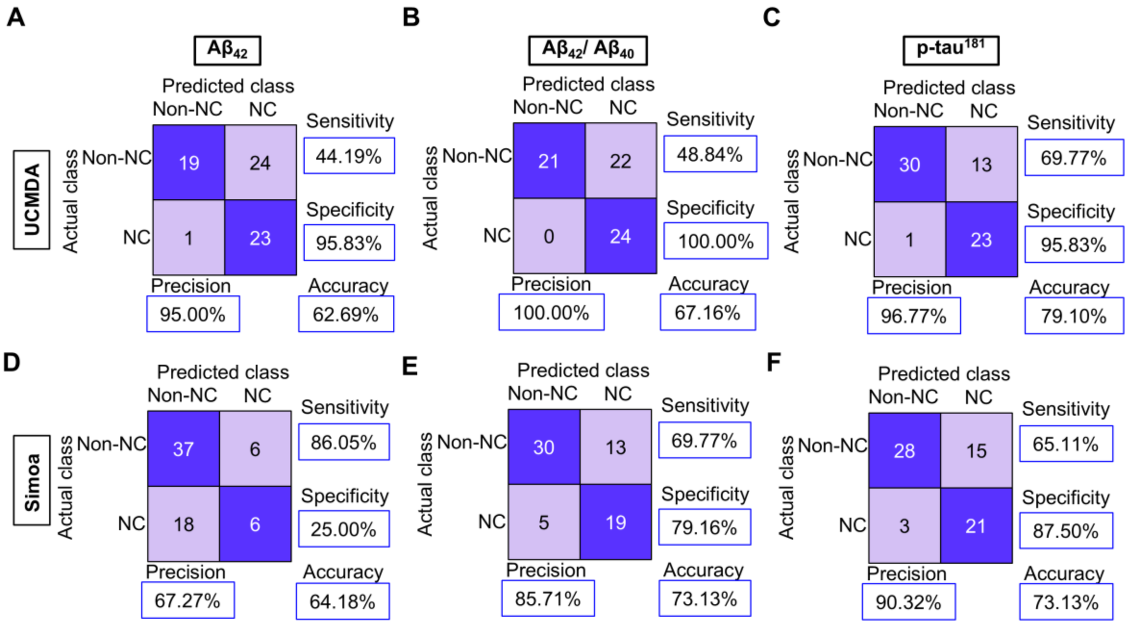

Figure S21 Comparison of the performance of UCMDA and Simoa in distinguishing non-NCs from NCs in the same clinical samples. (A-F) Comparison of the sensitivity, specificity, precision, and accuracy of UCMDA and Simoa in distinguishing non-NCs ($n = 43$) from NCs ($n = 24$) using the predictor of Aβ_{42} (A, D), Aβ_{42}/Aβ_{40} (B, E), or p-tau^{181} (C, F).

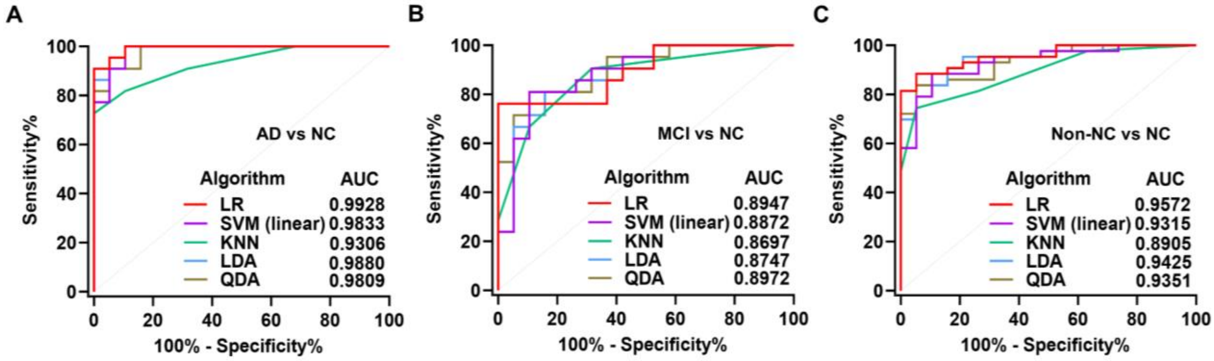

Figure S22 ROC curves of different algorithms for distinguishing AD, MCI, or non-NCs from NCs. (A-C) ROC curves of LR, SVM (linear), KNN, LDA, or QDA algorithm based on the combination of six biomarkers for distinguishing AD (A), AD-related MCI (B), or non-NCs (C) from NCs. (n = 22 for patients with AD, n = 21 for individuals with AD-related MCI, n = 19 for NCs, and n = 43 for non-NCs).

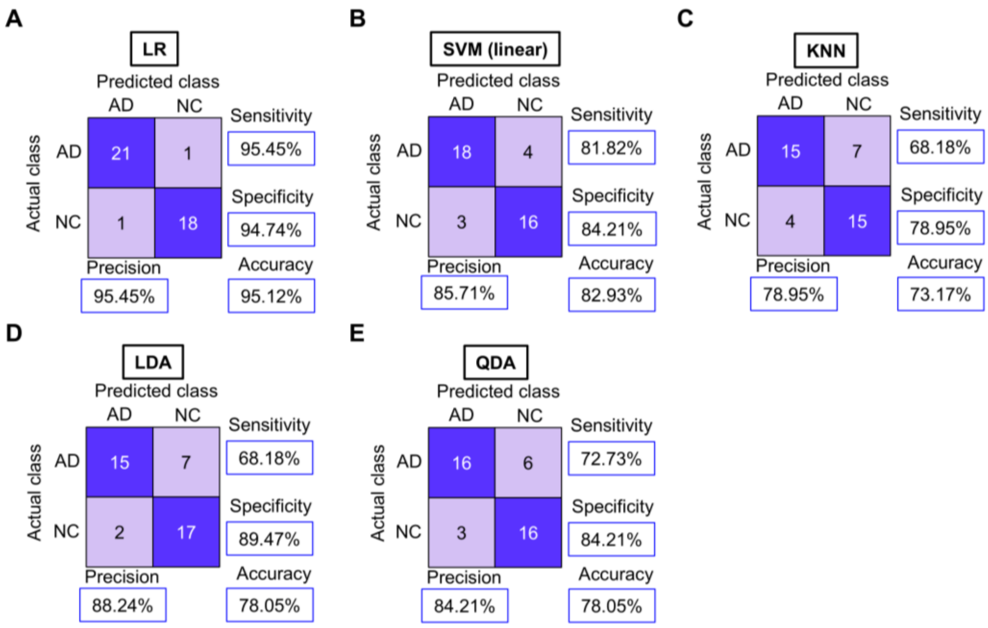

Figure S23 Comparison of the performance of different algorithms in distinguishing AD from NCs. (A-E) Confusion matrix and corresponding metrics (sensitivity, specificity, precision, and accuracy) of ROC analysis based on the LR (A), SVM (linear) (B), KNN (C), LDA (D), or QDA (E) algorithm using the predictor of six biomarkers detected by UCMDA to distinguish patients with AD (n = 22) from NCs (n = 19) in the test set.

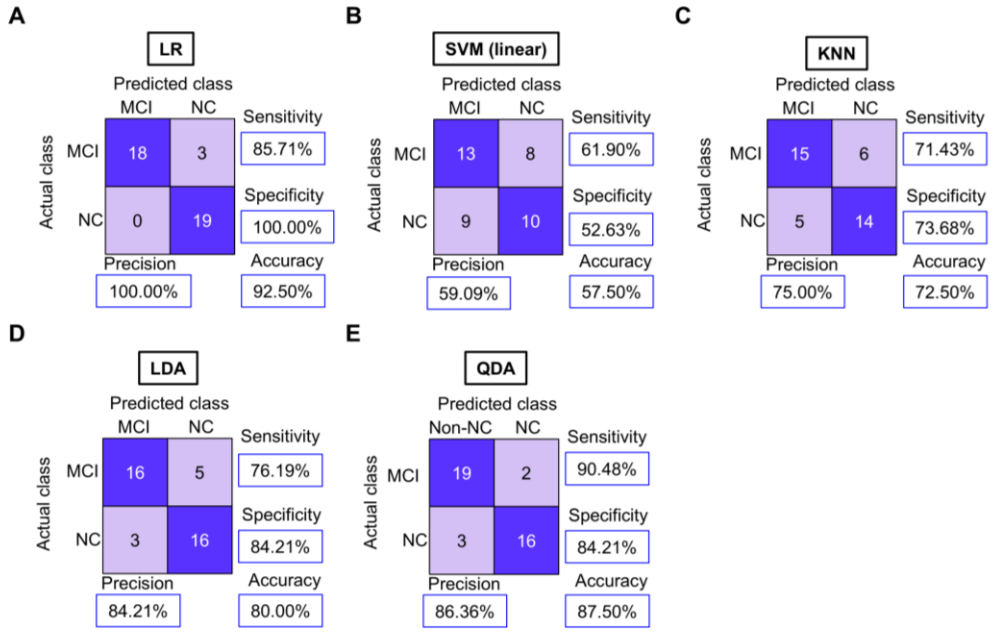

Figure S24 Comparison of the performance of different algorithms in distinguishing AD-related MCI from NCs. (A-E) Confusion matrix and corresponding metrics (sensitivity, specificity, precision, and accuracy) of ROC analysis based on the LR (A), SVM (linear) (B), KNN (C), LDA (D), or QDA (E) algorithm using the predictor of six biomarkers detected by UCMDA to distinguish individuals with AD-related MCI (n = 21) from NCs (n = 19) in the test set.

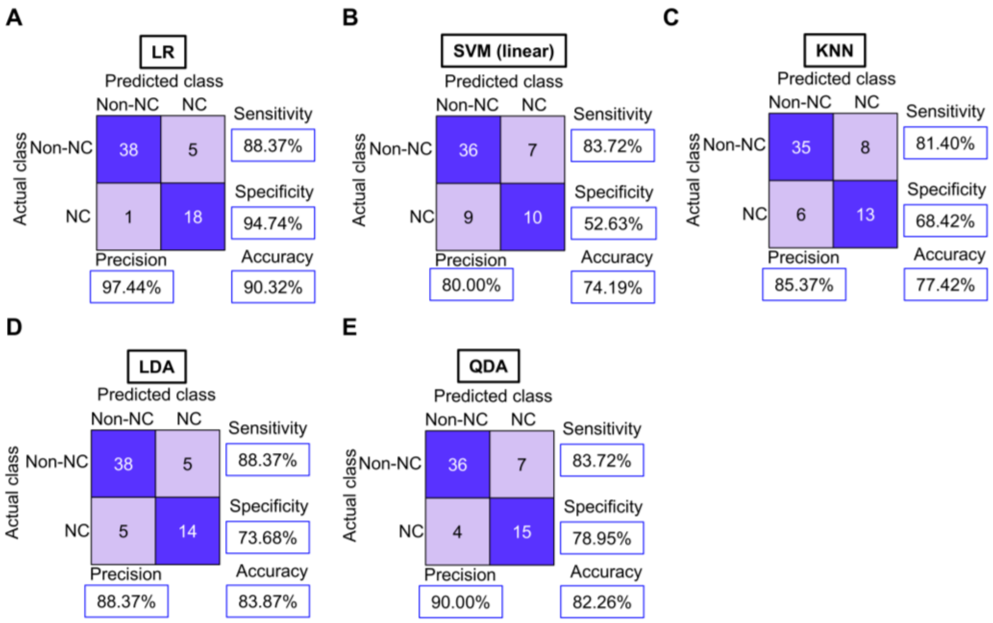

Figure S25 Comparison of the performance of different algorithms in distinguishing non-NCs from NCs. (A-E) Confusion matrix and corresponding metrics (sensitivity, specificity, precision, and accuracy) of ROC analysis based on the LR (A), SVM (linear) (B), KNN (C), LDA (D), or QDA (E) algorithm using the predictor of six biomarkers detected by UCMDA to distinguish non-NCs (n = 43) from NCs (n = 19).

Table S1. ssDNA sequences used in UCMDA

Name	Sequence (5'-3')
ssDNA 91	GGAGATTAGCAGTTTAGACTAAGAGTCCATTCCTCCCTCCTACT GCTATGGCTTAGGAAGTTCAGGTATAATTATGGGATCGTCATC GTTC
ssDNA 96	GCTACAAGGTTAATTAGGCAGTTACCAACCTGTTGATTTGGTAT AAGGCCAGAGGAATAATGCATAAGTGTGTCTCCAAGTGCTATA AACTTATGC
ssDNA 97	CAACTTAAAGGGGGATGTGTAGACTAGAAATATTTCCCCGAAG CCCATAAAGTACTATGATGCTGTATCTACTTTGCGTAGCAGATT TCTCTCAGAC
ssDNA 99	GACATACGGTCTTAATAGACCAAAGATTCGTGAGCGTAGTCCT GGGGGCTCGCCTACCGTTTAAAATAACCCAAAGTATAATCAAG GGGAACGCTCAAC
ssDNA O102	CTATGCTGTAACTTTCCCGGATGATATTTATGCGGAGAGCAGTA CTTCAGCGGATGCAGTAATTTCTGGTGACAATAACCCGGACCA TAAACTGAAAGTTTC
ssDNA N102	CAGCGCAATAATCTGGTTACTTTTCCTCAATTTTCTGATAGAAA CAATATCAGACAAAAGGAATATTATTTTCATTTTAATCAGATA ACCACTCTTCCGGAG

Table S2. RPA primer sequences used in UCMDA

Name	Sequence (5'-3')
ssDNA 91F	GGAGATTAGCAGTTTAGACTAAGAGTCCAT
ssDNA 91R	GAACGATGACGATCCCATAATTATACCTGA
ssDNA 96F	GCTACAAGGTTAATTAGGCAGTTACCAAC
ssDNA 96R	GCATAAGTTTATAGCACTTGGAGACACAC
ssDNA 97F	CAACTTAAAGGGGGATGTGTAGACTAGAA
ssDNA 97R	GTCTGAGAGAAATCTGCTACGCAAAGTA
ssDNA 99F	GACATACGGTCTTAATAGACCAAAGATTCG
ssDNA 99R	GTTGAGCGTTCCCCTTGATTATACTTTG
ssDNA O102F	CTATGCTGTAACTTTCCCGGATGATATTTA
ssDNA O102R	GAAACTTTCAGTTTATGGTCCGGGTTATT
ssDNA N102F	CAGCGCAATAATCTGGTTACTTTTCCTC
ssDNA N102R	CTCCGGAAGAGTGGTTATCTGATTAAAATG

Table S3. crRNA sequences and the probe used in UCMDA

Name	Sequence (5'-3')
crRNA 91	GAAUUUCUACUGUUGUAGAUGACUAAGAGUCCAUUCCUCCCUCC
crRNA 96	GAAUUUCUACUGUUGUAGAUGUAUAAGGCCAGAGGAAUAAUGC
crRNA 97	GAAUUUCUACUGUUGUAGAUCCCGAAGCCCAUAAAGUACUAUGA
crRNA 99	GAAUUUCUACUGUUGUAGAUAAAUAACCCAAAGUAUAAUCAAGG
crRNA O102	GAAUUUCUACUGUUGUAGAUUGCGGAGAGCAGUACUUCAGCGGA
crRNA N102	GAAUUUCUACUGUUGUAGAUUGAUAGAAACAAUAUCAGACAAAA
Probe	FAM-TTTTTT-BHQ

Table S4. Comparison of UCMDA with other detection methods

Method	Analytes	LOD	CV	AUC	Equipment	Ref
UCMDA	$A\beta_{40}$,<br>$A\beta_{42}$,<br>p-tau^{181},<br>p-tau^{217},<br>p-tau^{231},<br>p-tau^{396,404}	1 fg/mL	4% - 12.5%	0.5243,<br>0.8541,<br>0.8522,<br>0.9012,<br>0.8146,<br>0.7895	Simple	This work
Simoa	IL-21,<br>p-tau^{181},	1 fg/mL<br>9.31 pg/mL	< 10%	0.77	Complex	[1, 2]
MSD	IL-21,<br>p-tau^{181},<br>p-tau^{217},<br>or p-tau^{231}	0.8 pg/mL<br>0.13 pg/mL<br>0.04 pg/mL	< 10%	0.8,<br>0.81,<br>0.81	Complex	[2, 3]
Olink Proteomics	IL-8,<br>GDNF	9 fM<br>48 fM	< 10%	0.969-0.982	Complex	[4, 5]
Chemilum-inescence (Roche)	$A\beta_{42}$,<br>p-tau^{181}	100 pg/mL<br>8 pg/mL	1.3%-2.6%	NA	Complex	[6, 7]
ELISA	$A\beta_{42}$,<br>tau,<br>or p-tau^{181}	1.56 pg/mL	NA	0.93,<br>0.89,<br>0.88	Simple	[8]
lateral flow immunoassay	$A\beta_{42}$Ms,<br>$A\beta_{42}$Os	154 pg/mL	NA	NA	Simple	[9]
SERS based immunoassay	p-tau^{396,404},<br>tau,<br>$A\beta_{42}$	3.8 pg/mL<br>1.21 pg/mL<br>46.2 pg/mL	NA	0.93,<br>NA	Simple	[10, 11]
Immuno-PCR	$A\beta_{40}$,<br>$A\beta_{42}$,<br>p-tau^{181},<br>p-tau^{396,404}	10 fg/mL	NA	0.85,<br>0.993,<br>0.79,<br>0.95	Simple	[12]
Carbon Nanotubes array	$A\beta_{40}$,<br>$A\beta_{42}$,<br>tau,<br>p-tau	47.96 fg/mL<br>45.44 fg/mL<br>734.2 fg/mL<br>1.0 pg/mL	5.24%,<br>6.36%	0.608,<br>0.8,<br>0.815,<br>0.87	Simple	[13]

Table S5. Demographic characteristics of the participants

Participant information	Normal senile control	Mild cognitive impairment	AD
Numbers	47	52	56
Male/Female	22/25	24/28	32/24
Ages	68 ± 6.1	69.8 ± 6.1	70 ± 7.3
MoCA score	26.4 ± 2.2	22.5 ± 2.6	12.9 ± 5.2
CDR	0	0.42 ± 0.2	1.2 ± 0.6
Aβ-PET positive/negative	0/47	17/35	56/0

Table S6. AUC value, sensitivity, specificity, precision, and accuracy of ROC analysis based on UCMDA using the predictor of single biomarker in distinguishing between AD patients and NCs

Biomarker	AUC	Sensitivity	Specificity	Precision	Accuracy
$A\beta_{42}$	0.8514	66.07%	89.36%	88.10%	76.70%
$A\beta_{42}/A\beta_{40}$	0.8302	57.14%	100.00%	100.00%	76.70%
$p\text{-tau}^{181}$	0.8522	96.43%	61.70%	75.00%	80.58%
$p\text{-tau}^{217}$	0.9012	78.57%	85.11%	86.27%	81.55%
$p\text{-tau}^{231}$	0.8146	80.36%	74.47%	78.95%	77.67%
$p\text{-tau}^{396,404}$	0.7895	55.36%	93.62%	91.18%	72.82%

Table S7. AUC value, sensitivity, specificity, precision, and accuracy of ROC analysis based on UCMDA using the predictor of the single biomarker in distinguishing between AD-related MCI and NCs

Biomarker	AUC	Sensitivity	Specificity	Precision	Accuracy
$A\beta_{42}$	0.6833	38.46%	97.87%	95.24%	66.67%
$A\beta_{42}/A\beta_{40}$	0.6391	38.46%	100.00%	100.00%	67.68%
p-tau^{181}	0.7295	53.85%	82.98%	77.78%	67.68%
p-tau^{217}	0.7390	50.00%	89.36%	83.87%	68.69%
p-tau^{231}	0.6809	34.62%	95.74%	90.00%	63.64%
p-tau^{396,404}	0.6653	48.08%	76.60%	69.44%	61.62%

Table S8. AUC value, sensitivity, specificity, precision, and accuracy of ROC analysis based on UCMDA using the predictor of single biomarker in distinguishing between non-NCs and NCs

Biomarker	AUC	Sensitivity	Specificity	Precision	Accuracy
$A\beta_{42}$	0.7719	51.85%	93.62%	94.94%	64.52%
$A\beta_{42}/A\beta_{40}$	0.7382	48.15%	100.00%	100.00%	63.87%
p-tau^{181}	0.7931	79.63%	65.96%	84.31%	75.48%
p-tau^{217}	0.8231	66.67%	85.11%	91.14%	72.26%
p-tau^{231}	0.7502	61.11%	78.72%	86.84%	66.45%
p-tau^{396,404}	0.7297	42.59%	93.62%	93.88%	58.06%

Table S9. Comparison of AUC value, sensitivity, specificity, precision, and accuracy of UCMDA and Simoa using the predictor of $A\beta_{42}$, $A\beta_{42}/A\beta_{40}$, or p-tau[181] in distinguishing between AD patients and NCs

Methods	Biomarker	AUC	Sensitivity	Specificity	Precision	Accuracy
UCMDA	$A\beta_{42}$	0.765	71.43%	70.83%	58.82%	71.05%
	$A\beta_{42}/A\beta_{40}$	0.842	57.14%	100.00%	100.00%	84.21%
	p-tau[181]	0.982	92.86%	95.83%	92.86%	94.74%
Simoa	$A\beta_{42}$	0.659	71.43%	62.50%	52.63%	65.79%
	$A\beta_{42}/A\beta_{40}$	0.833	78.57%	79.17%	68.75%	78.95%
	p-tau[181]	0.967	92.86%	87.50%	81.25%	89.47%

Table S10. Comparison of AUC value, sensitivity, specificity, precision, and accuracy of UCMDA and Simoa using the predictor of $A\beta_{42}$, $A\beta_{42}/A\beta_{40}$, or p-tau^{181} in distinguishing between AD-related MCI and NCs

**Methods**	**Biomarker**	**AUC**	**Sensitivity**	**Specificity**	**Precision**	**Accuracy**
UCMDA	$A\beta_{42}$	0.649	44.83%	95.83%	92.86%	67.92%
	$A\beta_{42}/A\beta_{40}$	0.701	44.83%	100.00%	100.00%	69.81%
	p-tau^{181}	0.810	58.62%	95.83%	94.44%	75.47%
Simoa	$A\beta_{42}$	0.566	62.07%	62.50%	66.67%	62.26%
	$A\beta_{42}/A\beta_{40}$	0.708	65.52%	79.17%	79.17%	71.70%
	p-tau^{181}	0.746	65.52%	75.00%	76.00%	69.81%

Table S11. Comparison of AUC value, sensitivity, specificity, precision, and accuracy of UCMDA and Simoa using the predictor of $A\beta_{42}$, $A\beta_{42}/A\beta_{40}$, or p-tau^{181} in distinguishing between non-NCs and NCs

Methods	Biomarker	AUC	Sensitivity	Specificity	Precision	Accuracy
UCMDA	$A\beta_{42}$	0.687	44.19%	95.83%	95.00%	62.69%
	$A\beta_{42}/A\beta_{40}$	0.747	48.84%	100.00%	100.00%	67.16%
	p-tau^{181}	0.866	69.77%	95.83%	96.77%	79.10%
Simoa	$A\beta_{42}$	0.507	86.05%	25.00%	67.27%	64.18%
	$A\beta_{42}/A\beta_{40}$	0.749	69.77%	79.16%	85.71%	73.13%
	p-tau^{181}	0.818	65.11%	87.50%	90.32%	73.13%

Table S12. The AUC value, sensitivity, specificity, precision, and accuracy of different algorithms using the predictor of composite biomarkers detected by UCMDA in distinguishing between AD patients and NCs

**Algorithms**	**AUC**	**Sensitivity**	**Specificity**	**Precision**	**Accuracy**
LR	0.9928	95.45%	94.74%	95.45%	95.12%
SVM (linear)	0.9833	81.82%	84.21%	85.71%	82.93%
KNN	0.9306	68.18%	78.95%	78.95%	73.17%
LDA	0.9880	68.18%	89.47%	88.24%	78.05%
QDA	0.9809	72.73%	84.21%	84.21%	78.05%

Table S13. The AUC value, sensitivity, specificity, precision, and accuracy of different algorithms using the predictor of composite biomarkers detected by UCMDA in distinguishing between AD-related MCI and NCs

**Algorithms**	**AUC**	**Sensitivity**	**Specificity**	**Precision**	**Accuracy**
LR	0.8947	85.71%	100.00%	100.00%	92.50%
SVM (linear)	0.8872	61.90%	52.63%	59.09%	57.50%
KNN	0.8697	71.43%	73.68%	75.00%	72.50%
LDA	0.8747	76.19%	84.21%	84.21%	80.00%
QDA	0.8972	90.48%	84.21%	86.36%	87.50%

Table S14. The AUC value, sensitivity, specificity, precision, and accuracy of different algorithms using the predictor of composite biomarkers detected by UCMDA in distinguishing between non-NCs and NCs

Algorithms	AUC	Sensitivity	Specificity	Precision	Accuracy
LR	0.9572	88.37%	94.74%	97.44%	90.32%
SVM (linear)	0.9315	83.72%	52.63%	80.00%	74.19%
KNN	0.8905	81.40%	68.42%	85.37%	77.42%
LDA	0.9425	88.37%	73.68%	88.37%	83.87%
QDA	0.9351	83.72%	78.95%	90.00%	82.26%

Table S15. The weight of each biomarker in the composite signature of LR

Group	(Intercept)	$A\beta_{42}$	$A\beta_{40}$	$A\beta_{42}/A\beta_{40}$
AD vs NC	1.425e+01	1.721e-05	5.127e-04	1.007e+02
AD-MCI vs NC	4.359e+00	2.436e-04	-4.555e-04	-4.899e+00
Non-NC vs NC	5.455e-01	3.386e-03	-3.988e-06	-4.985e+00

Group	$p\text{-}tau^{181}$	$p\text{-}tau^{217}$	$p\text{-}tau^{231}$	$p\text{-}tau^{396,404}$
AD vs NC	-7.558e-08	-1.377e-04	-5.751e-03	2.875e-06
AD-MCI vs NC	-7.177e-07	-8.568e-06	-2.561e-02	-1.253e-05
Non-NC vs NC	-4.141e-05	-7.975e-06	-6.256e-05	-2.575e-02

Table S16. Details of antibodies used in UCMDA platform

Antibody name	Target	Catalog number	Species	Source
A40	$A\beta_{40}$	V28704	Mouse	Commercial purchase
6E8	$A\beta_{42}$	V28702	Mouse	Commercial purchase
1F12	$A\beta_{42}$	1F12	Mouse	Laboratory preparation
2C6	$A\beta_{42}$	2C6	Mouse	Laboratory preparation
1G11	Total tau	1G11	Mouse	Laboratory preparation
1E9	p-tau^{181}	1E9	Mouse	Laboratory preparation
1A2	p-tau^{217}	1A2	Mouse	Laboratory preparation
2H2	p-tau^{231}	2H2	Mouse	Laboratory preparation
4B1	p-tau^{396,404}	4B1	Mouse	Laboratory preparation

Table S17. The sequence of synthesized peptides used in this study

**Name**	**Sequence (N'-C')**
$A\beta_{38}$	DAEFRHDSGYEVHHQKLVFFAEDVGSNKGAIIGLMVGG
$A\beta_{40}$	DAEFRHDSGYEVHHQKLVFFAEDVGSNKGAIIGLMVGGVV
$A\beta_{42}$	DAEFRHDSGYEVHHQKLVFFAEDVGSNKGAIIGLMVGGVVIA
p-tau^{181}	AKTPPAPK(p-T)PPSSGEP
p-tau^{217}	SRTPSLP(p-T)PPTREPK
p-tau^{231}	KVAVVR(p-T)PPKSPS
p-tau^{396,404}	RENAKAKTDHGAEIVYK(p-S)PVVSGDT(p-S)PRHL
Tau-p-tau^{181}	SGYSSPGSPGTPGSRSRTPSLPTPPTGGGGSAKTPPAPK(p-T)PPSSGEP
Tau-p-tau^{396,404}	SGYSSPGSPGTPGSRSRTPSLPTPPTGGGGSRENAKAKTDHGAEIVYK(p-S)PVVSGDT(p-S)PRHL

References

1. Lleó, A., H. Zetterberg, J. Pegueroles, et al., "Phosphorylated tau181 in plasma as a potential biomarker for Alzheimer's disease in adults with Down syndrome", *Nature Communications* **12** (2021): 4304, https://doi.org/10.1038/s41467-021-24319-x.
2. Poorbaugh, J., T. Samanta, S.W. Bright, et al., "Measurement of IL-21 in human serum and plasma using ultrasensitive MSD S-PLEX® and Quanterix SiMoA methodologies", *Journal of Immunological Methods* **466** (2019): 9-16, https://doi.org/10.1016/j.jim.2018.12.005.
3. Mielke, M.M., R.D. Frank, J.L. Dage, et al., "Comparison of Plasma Phosphorylated Tau Species With Amyloid and Tau Positron Emission Tomography, Neurodegeneration, Vascular Pathology, and Cognitive Outcomes", *JAMA Neurology* **78** (2021): 1108-1117, https://doi.org/10.1001/jamaneurol.2021.2293.
4. Lundberg, M., A. Eriksson, B. Tran, et al., "Homogeneous antibody-based proximity extension assays provide sensitive and specific detection of low-abundant proteins in human blood", *Nucleic Acids Research.* **39** (2011): e102, https://doi.org/10.1093/nar/gkr424.
5. Mattingly, Z.A., A.M. Celia, Y.-k. Kuo, et al., "Technical performance and biotemporal stability evaluation of Olink proximity extension assay for blood-based biomarker discovery", *Alzheimer's & Dementia* **17** (2021): e056318, https://doi.org/https://doi.org/10.1002/alz.056318.
6. Hansson, O., J. Seibyl, E. Stomrud, et al., "CSF biomarkers of Alzheimer's disease concord with amyloid-β PET and predict clinical progression: A study of fully automated immunoassays in BioFINDER and ADNI cohorts", *Alzheimer's & Dementia* **14** (2018): 1470-1481, https://doi.org/10.1016/j.jalz.2018.01.010.
7. Rutz, S., E. Manuilova, L. Müller-Hübner, et al., "Second-generation fully automated Elecsys cerebrospinal fluid immunoassays demonstrate high precision, reproducibility, and sample stability suitable for clinical use to aid Alzheimer's disease diagnosis", *Alzheimer's & Dementia* **18** (2022): e069299, https://doi.org/https://doi.org/10.1002/alz.069299.
8. Jia, L., Q. Qiu, H. Zhang, et al., "Concordance between the assessment of Aβ42, T-tau, and P-T181-tau in peripheral blood neuronal-derived exosomes and cerebrospinal fluid", *Alzheimer's & Dementia* **15** (2019): 1071-1080, https://doi.org/10.1016/j.jalz.2019.05.002.

9. Zhang, L., X. Du, Y. Su, et al., "Quantitative assessment of AD markers using naked eyes: point-of-care testing with paper-based lateral flow immunoassay", *Journal of Nanobiotechnology* **19** (2021): 366, https://doi.org/10.1186/s12951-021-01111-z.

10. Zhang, L., Y. Su, X. Liang, et al., "Ultrasensitive and point-of-care detection of plasma phosphorylated tau in Alzheimer's disease using colorimetric and surface-enhanced Raman scattering dual-readout lateral flow assay", *Nano Research* **16** (2023): 7459-7469, https://doi.org/10.1007/s12274-022-5354-4.

11. Zhang, X., S. Liu, X. Song, et al., "Robust and Universal SERS Sensing Platform for Multiplexed Detection of Alzheimer's Disease Core Biomarkers Using PAapt-AuNPs Conjugates", *Acs Sensors* **4** (2019): 2140-2149, https://doi.org/10.1021/acssensors.9b00974.

12. Hu, S., L. Zhang, Y. Su, et al., "Sensitive detection of multiple blood biomarkers via immunomagnetic exosomal PCR for the diagnosis of Alzheimer's disease", *Science Advances* **10** (2024): eabm3088, https://doi.org/10.1126/sciadv.abm3088.

13. Kim, K., M.J. Kim, D.W. Kim, et al., "Clinically accurate diagnosis of Alzheimer's disease via multiplexed sensing of core biomarkers in human plasma", *Nature Communications* **11** (2020): 119, https://doi.org/10.1038/s41467-019-13901-z.
